# Mitochondrial dysfunction and oxidative stress in autism spectrum disorder: pharmacological insights into natural antioxidants

**DOI:** 10.3389/fphar.2026.1783888

**Published:** 2026-04-15

**Authors:** Marta Sofia Scenna, Erjola Bej, Patrizia Cesare, Ilaria Persichitti, Annamaria Cimini, Michele D’Angelo, Vanessa Castelli

**Affiliations:** 1 Laboratory of Neuropharmacology and Applied pharmacology, Department of Life, Health and Environmental Sciences, University of L'Aquila, L’Aquila, Italy; 2 Department of the Chemical-Toxicological and Pharmacological Evaluation of Drugs, Faculty of Pharmacy, Catholic University Our Lady of Good Counsel, Tirana, Albania

**Keywords:** autism, crocin, curcumin, electron transport chain, quercetin, resveratrol, safranal, vitamins

## Abstract

Autism spectrum disorder (ASD) is a group of neurodevelopmental disorders characterized by social communication deficits, restricted and fixated interests and abnormal motor behaviors. Increasing evidence implicates oxidative stress, mitochondrial dysfunction, and neuroinflammation as key biological features of ASD. Aberrant redox homeostasis, reduced glutathione reserves, increased lipid peroxidation, and dysregulated NRF2 signaling have been documented in both peripheral tissues and brain samples. Post-mortem and imaging studies further reveal deficits in electron transport chain complexes and pyruvate dehydrogenase activity, suggesting a mechanistic link between mitochondrial bioenergetics and ASD-related phenotypes. These pathomechanisms have motivated interest in antioxidant metabolites from botanical drugs and nutrients as complementary strategies. To critically appraise mechanisms and levels of evidence (*in vitro, in vivo*, clinical) for vitamin E and C, glutathione and its precursors, polyphenols (quercetin, resveratrol, curcumin), *Crocus sativus* carotenoids (crocin/safranal), and “indirect” modulators (e.g., omega-3, folinic acid), emphasizing study quality, translational relevance, and limitations. The aim of this review is to synthesize current findings on the potential benefits of antioxidants in addressing both molecular and behavioral aspects of ASD, while also examining the link between oxidative stress and ASD. Furthermore, we discuss the role of antioxidant-based interventions in managing ASD symptoms. The review highlights the complex challenges associated with antioxidant therapies and deficiencies, emphasizing the need for a multifaceted nutritional approach particularly in children with ASD.

## 1 Introduction

**FIGURE 1:**
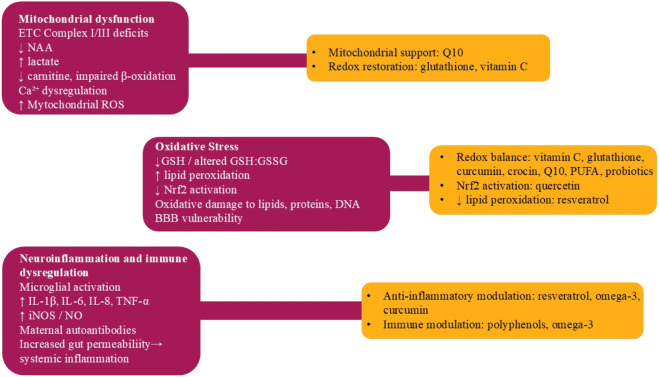
ASD pathophysiology and antioxidant points of action. Schematic of oxidative stress, mitochondrial dysfunction (ETC deficits/ROS), BBB/glial responses, and points of action for vitamin C/E, glutathione/precursors, resveratrol, curcumin, crocin/safranal; link to NRF2 and redox homeostasis.

Autism spectrum disorder (ASD) is a lifelong neurodevelopmental disorder characterized by deficits in social communication and interaction, as well as restricted and repetitive patterns of behavior, interests, and activities, often accompanied by atypical sensory-motor responses (Lord et al., 2018). Language development is frequently affected, encompassing delayed acquisition and severe impairments in expressive abilities. In many cases, ASD co-occurs with intellectual disability, further complicating cognitive and adaptative functioning (Faja and Dawson, 2017). ASD typically emerges within the first 3 years of life, during which affected toddlers often show difficulties expressing emotions, establishing social connection, and developing age-appropriate relationships. Furthermore, the heterogeneity and complexity of ASD reflect both in the variability of behavioral profiles among affected individuals and in the behavioral changes that may occur within the same individual over time. Repetitive movements observed in early childhood may shift toward different types of stereotyped patterns during adolescence, although the repetitive and restricted nature of the action typically persists. An increasing body of evidence suggests that these features may be modulated by sex-related differences. While earlier research suggested few significant distinctions, more recent findings indicate that females may exhibit more severe mood disorders and anxiety, and are often diagnosed later due to subtler symptom profiles and higher camouflaging abilities (Dellapiazza et al., 2022; Napolitano et al., 2022) (Figure 1).

ASD results from a complex interplay between genetic and environmental factors. Genetically, ASD can be linked to identifiable genetic syndromes, such as Fragile X, Tuberous Sclerosis, Neurofibromatosis I, ADNP syndrome, and copy number variations (Lubbers et al., 2024; Salcedo-Arellano et al., 2021). In addition, it can also be associated with other medical conditions including epilepsy, congenital cardiac defects, gastrointestinal (GI), immunological, endocrine/metabolic and sleep disorders, as well as psychiatric comorbidities like ADHD and anxiety. The role of environmental factors in ASD became evident with the observation that advanced paternal and maternal age is associated with an increased likelihood of having a child with ASD (Croen et al., 2007). Moreover, prospective studies have reported increased cases of autoimmune diseases in families of children with ASD, especially in mothers (Ashwood and Van De Water, 2004). During the prenatal period, most of the brain anomalies that are linked to ASD take place, therefore, environmental exposure to teratogen compounds, such as valproic acid (VPA) and thalidomide, may adversely affect fetal neurodevelopment (Arndt et al., 2005). During the perinatal period, a study conducted by Badawi and colleagues reported that 5% of newborn survivors with encephalopathy were later diagnosed with ASD (Badawi et al., 2006). Neuropathological and imaging studies report macrostructural and microstructural brain differences, including reduced Purkinje cells (Johnson et al., 2007), neocortical anomalies, macrocephaly in early life, and altered trophic signaling, all consistent with atypical neurodevelopmental trajectories (Courchesne et al., 2001; McCaffery and Deutsch, 2005). One of the possible mechanisms thought to be related to brain overgrowth is the increased blood concentrations of brain-derived neurotrophic factor (BDNF) in newborns who were later diagnosed with ASD (Hazlett et al., 2011; Nelson et al., 2001).

Converging evidence highlights mitochondrial dysfunction and oxidative stress, resulting from excessive production of reactive oxygen species (ROS) and/or insufficient antioxidant defenses, in both peripheral tissues and brain samples of individuals with ASD (Chełchowska et al., 2025; Davinelli et al., 2025; Lin et al., 2022) This oxidative dysregulation is thought to exacerbate neuroinflammation, mitochondrial dysfunction, and synaptic abnormalities, which may underlie some of the core behavioral and cognitive impairments seen in ASD (Gevezova et al., 2023).

In this biological context, natural compounds with antioxidant and anti-inflammatory properties have attracted growing interest as complementary strategies to modulate redox homeostasis, immune signaling, and mitochondrial function in ASD. Therefore, this review aims to provide an overview of the potential advantages of antioxidant compounds in influencing both the molecular mechanisms and behavioral characteristics associated with ASD. We summarize mechanistic insights across cellular and molecular domains, critically appraise preclinical and clinical findings, and discuss translational challenges that include bioavailability and brain penetration, study heterogeneity, biomarker guided stratification, and pediatric safety. Finally, we outline future directions within an ethnopharmacological perspective, integrating traditional knowledge with pharmacological innovation to inform multi target, standardized, and potentially personalized interventions for defined ASD endophenotypes.

## 2 Mitochondrial dysfunction in ASD

From a clinical point of view, mitochondrial dysfunction in ASD may be the cause of seizures, motor delay, fatigue and neurodevelopmental regression (Frye et al., 2024) These impairments may arise from genetic irregularities, including chromosomal abnormalities and mutations in both mitochondrial DNA (mtDNA) and nuclear genes that are involved in mitochondrial function (Valiente-Pallejà et al., 2018) Mitochondrial dysfunction in ASD can be found in fibroblasts, gastrointestinal, muscle and brain cells and it is probably caused by toxicants and microbiome metabolites that can alter and modulate its normal function (Rose et al., 2018). This impairment compromises mitochondrial ATP production, resulting in cellular energy deficits (Siddiqui et al., 2016).

Mitochondrial dysfunction itself can be categorized into two types: the first type represents a mutation of a gene which is associated with the ATP-generating pathway, while the second one is related to various abnormalities such as genetic, biochemic or metabolic, that may weaken the ability of the mitochondria to produce ATP (Haas et al., 2007). However, recent studies suggest a shift from this traditional classification toward the identification of distinct mitochondrial phenotypes in ASD. These include subgroups with hyperactive mitochondrial respiration, partial disfunction, or classical mitochondrial disease patterns, each potentially associated with different clinical features such as neurodevelopmental regression or immune malfunction (Frye et al., 2024).

**FIGURE 2:**
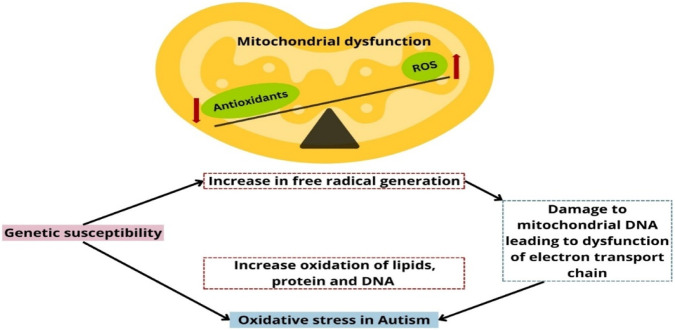
Mitochondrial dysfunction as a possible cause of oxidative stress in ASD.

A central mechanism often implicated is the dysfunction of Electron Transport Chain (ETC), which plays a crucial role in ATP production. Impairments in the ETC can lead to electron leakage and the excessive generation of reactive oxygen species (ROS). When ROS levels exceed those of antioxidant defenses, there is an aberrant increase in free radical generation resulting in mitochondrial damage and leading to oxidative stress and subsequently this increased stress can reduce ETC activity (Gu et al., 2013) (Figure 2).

This oxidative stress environment can activate immune signalling pathways promoting neuroinflammation through mitochondrial dysfunction and impaired mitophagy, as observed in recent studies on ASD brains (Pinto Payares et al., 2024). Notably, post-mortem studies revealed reduced activity of respiratory chain complexes in a group of ASD children, suggesting that abnormalities, especially in the frontal, temporal and cerebellar areas (Chauhan et al., 2011; Tang et al., 2013), may cause mitochondrial impairment and contribute to the appearance of the first symptoms (Siddiqui et al., 2016). In these areas, studies reported reduced activity of non-ETC mitochondrial enzymes, such as aconitase and pyruvate dehydrogenase. Supporting these findings, a recent PET (positron emission tomography) imaging study by (Kato et al., 2023) demonstrated a significant reduction in Complex I availability in the anterior cingulate cortex of ASD individuals, reinforcing the hypothesis that compromised mitochondrial respiration plays a key role in ASD pathophysiology. Furthermore, ETC deficiencies are noted in the subunits that are encoded by the mitochondrial DNA, specifically in the ETC Complex III (Poling et al., 2006).

Other post-mortem studies revealed mitochondrial dysfunction and altered calcium ( ${Ca}^{2+}$ ) signaling. One line of the research is that mutations arrest or block voltage-dependent channel inactivation resulting in ${Ca}^{2+}$ influx. Other mechanisms like mutations that enhance cytosolic ${Ca}^{2+}$ levels or intensify intracellular ${Ca}^{2+}$ signaling have also been associated with ASD (Krey and Dolmetsch, 2007). Mitochondria play a crucial role in managing the cellular ${Ca}^{2+}$ , andincreased ${Ca}^{2+}$ levels could activate an immune response similar to that detected in ASD brains (Larbi et al., 2007).

**FIGURE 3:**
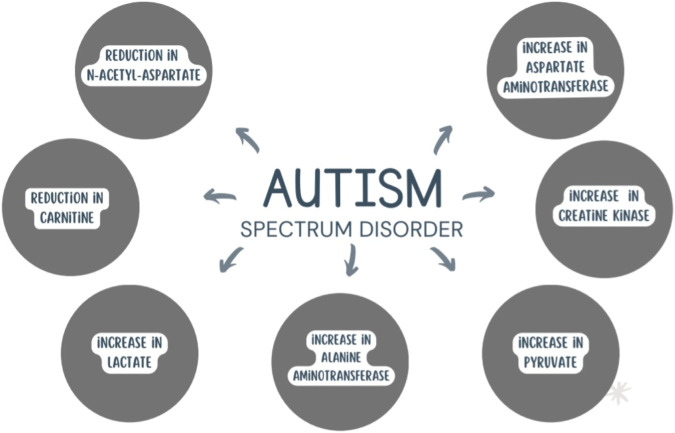
Metabolic biomarkers altered in ASD.

Mitochondrial dysfunction in ASD is further supported by the presence of non-specific biomarkers including reduction in carnitine and increases in lactate, pyruvate, creatine kinase, aspartate aminotransferase and alanine aminotransferase compared to control groups (Frye, 2020; Rossignol and Frye, 2012). Furthermore, one of the most important brain metabolic markers is N-acetyl-aspartate (NAA) which contributes to the production of energy by the mitochondria. Studies conducted on a group of children affected by ASD revealed a reduction of the NAA concentrations (Friedman et al., 2003) (Figure 3).

Another notable finding revealed by PET and NMR (nuclear magnetic resonance) spectroscopy analysis, is that in the brains of patients affected by ASD were found decreased glucose utilization and reduced ATP levels, further confirming mitochondrial dysfunction (Palmieri and Persico, 2010).

Other studies reported lactic acidosis and increased alanine levels, it is highly recognized that high concentrations of lactate can inhibit mitochondrial respiration and disbalance the redox equilibrium (Oliveira et al., 2005). This hypothesis is supported by post-mortem evidence of reduced pyruvate dehydrogenase activity in the brains of individuals with ASD (Gu et al., 2013), which may impair the conversion of pyruvate to acetyl-CoA, leading to mitochondrial dysfunction and accumulation of lactate and alanine. Therefore, the reduction of its activity can cause insufficient ATP production.

Alongside impairments in carbohydrate metabolism, evidence of reduced carnitine levels suggest a dysfunction in mitochondrial β-oxidation, leading to accumulation of long-chain fatty acids and elevated markers of lipid elongation and desaturation (Filipek et al., 2004; Pastural et al., 2009). This metabolic imbalance contributes to increased lipid peroxidation, as indicated by high malondialdehyde (MDA) levels (Chauhan et al., 2004), and is exacerbated by a concomitant reduction in antioxidant defenses.

These mitochondrial impairments not only compromise energy production but also act as pro-inflammatory stimuli, promoting chronic neuroinflammation through dysregulated mitochondrial signaling and innate immune response.

## 3 Oxidative stress and ASD

As mentioned before, oxidative stress can be the cause of cellular damage through ROS or reactive nitrogen species (RNS). If ROS levels are moderately low, cells are able to respond adequately to oxidative stress, this condition is called eustress. On the other hand, in case of excessive distress, cells are exposed to high levels of ROS which can cause neuroinflammation, damage to the blood-brain barrier (BBB) and impairment to astrocyte crosstalk leading to ASD (El-Ansary et al., 2018; Russo et al., 2018).

The pathogenesis of ASD might be connected to the gathering of oxidized products and the disorder of the redox homeostasis (Frustaci et al., 2012; Raymond et al., 2014). Under physiological conditions, the BBB serves as a critical interface composed of endothelial cells connected by tight junction proteins, pericytes, and surrounding glial elements regulating the transport of molecules to the central nervous system. However, the brain is highly susceptible to oxidative damage due to its high oxygen consumption and limited antioxidant defenses (Sies et al., 2017). Indeed, excessive ROS disrupt the BBB integrity, impairing its selective permeability (Hu et al., 2020). This disruption not only compromises transport processes but may also allow the infiltration of pro-inflammatory cytokines and autoantibodies (*i.e.*, maternal IgG) into the fetal brain during development (Braunschweig and Van De Water, 2012; Croen et al., 2008). Even though the human brain weighs less than 2% of the body mass, it is the organ that consumes the most oxygen and therefore, it is more susceptible to oxidative stress (Sies et al., 2017). Since glutathione levels are lower from conception to infancy, children are even more at risk of oxidative stress. This cascade of oxidative damage and barrier dysfunction is increasingly recognized as a core mechanism linking neuroinflammation to the neurodevelopmental abnormalities observed in ASD (El-Ansary et al., 2018; Russo et al., 2018).

In the brain of children with ASD, low levels of mitochondrial glutathione, mitochondrial dysfunction, oxidative stress and oxidative damage to macromolecules such as lipids, proteins and DNA have been reported (James et al., 2009). ROS interaction with these macromolecules can cause lipid peroxidation, gene mutation, enzyme inactivation and protein denaturation (Nathan and Cunningham-Bussel, 2013; Sena and Chandel, 2012). Oxidative stress also causes inflammatory response and consequently, ASD patients are reported to have immunological abnormalities and an activated inflammatory response (Prata et al., 2019; Vargas et al., 2005). Moreover, oxidative stress can also be the cause of neurodevelopmental disorder, cerebral injury and neuro dysfunction, all leading to ASD (Manivasagam et al., 2020; Pisoschi and Pop, 2015). Interestingly, the SOD activity in the red blood cells was elevated in ASD children if compared to the control group and that is probably because this mechanism acts against the cell damage caused by the oxidative stress in the brain (Pangrazzi et al., 2020a). Furthermore, studies have reported an aberrant activation of NRF2, a key antioxidant transcription factor involved in the regulation of critical antioxidants including glutathione, SOD, and CAT, leading to reduced antioxidant capacity (Davinelli et al., 2025) In ASD children, the oxidative stress is not only higher, but it manifests in a complicated new pattern concerning different types of ROS and antioxidants (Hirayama et al., 2020).

## 4 Immune dysregulation and neuroinflammation in ASD

ASD itself is related to altered neuroinflammatory mechanisms and unusual immune responses. The brain is constituted by a preponderance of microglia and astrocytes, these glial cells are essential in the Central Nervous System (CNS), with microglia functioning as innate immune cells involved in surveillance and defense, while astrocytes modulate neuronal communication, maintain the extracellular environment, and regulate inflammatory signaling (Garland et al., 2022) Both brain cells are able to produce pro- and anti-inflammatory cytokines (Schwarz and Bilbo, 2012). The activation of astrocytes and microglia plus the regulation of cytokine activity is strictly related to ASD (Patterson, 2009). Indeed, microglial activation leads to increased production of cytokines and activation of nitric oxide synthases (iNOS). The enhancement of iNOS increases the nitric oxide which has harmful and dangerous effects on neurons (Fernandes et al., 2014; Takano, 2015). According to studies on rodents, high levels of cytokines might be the cause of the characteristic symptoms of behaviour present in both development and adulthood in ASD (Depino, 2013). The use of PET indicated that there was an activation of the microglia in both ASD and schizophrenia but although some of the inflammatory markers are common, such as IL-6, IL-8 and TNF-α, the scientists found a difference in IL-1β which was mostly found in schizophrenia patients (Bloomfield et al., 2016; Li et al., 2009). In ASD serotonin and IL-6 have been proposed as potential combined biomarkers since both present increased levels compared to control groups (Yang et al., 2015).

Multiple studies have shown elevated levels of cytokine in peripheral blood of children with ASD. Ashwood et al. (Ashwood et al., 2011), observed significantly higher level of cytokines, specifically IL-1β, IL-6, IL-8, IL-12p40,in the plasma of 97 children aged 2-5, if compared to the levels of age-matched children of the control group or children with other developmental conditions. Enstrom et al., 2010 found elevated secretion of pro-inflammatory cytokines, IL-6, IL-1β, TNFαfrom cultured peripheral blood monocytes of ASD children cultured and stimulated *in vitro* (Enstrom et al., 2010). Furthermore, cytokine profile have been correlated with ASD severity: in children with moderate to mild ASD, IL-12p40 were found to be increased while higher levels of tumor necrosis factor alpha (TNF-α) were found in children with moderate severity (Inga Jácome et al., 2016). Severe ASD has been linked to elevated levels of IL-1β and IL-4 in the neonatal blood (Theoharides et al., 2016), and increased serum levels of IL-1β and IL-6 compared to control groups (Emanuele et al., 2010). Notably, higher interleukin concentrations were associated with more severe behavioral symptoms (Ashwood et al., 2011).

Recent data support findings that immune cells activity in ASD subjects is compromised, with amplified inflammatory signaling and reactivity of immune cells such as B-cells, neutrophils and monocytes (Majerczyk et al., 2022). T-cells are altered in ASD patients and the equilibrium between the different phenotypes is important in the progression of the disorder (Ellul et al., 2021). A great amount of Th17cell phenotype has been linked to ASD pathogenesis (Moaaz et al., 2019). After exposure to immunostimulants, there is an enhancement of cytokines in the fetal brain and maternal serum, amniotic fluid and placenta (Urakubo et al., 2001). One hypothesis suggests that the dysregulation of maternal immune system itself during pregnancy could be a possible cause of ASD. In cases of maternal autoimmunity, IgG antibodies of the mother enter the fetal chamber crossing the placenta. The IgG can interfere with the fetal development and inhibit the complete formation of the BBB of the fetus (Braunschweig and Van De Water, 2012; Croen et al., 2008).

Evidence suggests that brain inflammation from pregnancy till a year after birth might contribute to ASD (Theoharides et al., 2013). In case of autoimmune components, auto-antibodies directed at fetal brain proteins might cause BBB damage (Braunschweig and Van De Water, 2012; Zimmerman et al., 2007). Maternal autoimmune diseases have been suggested as a trigger for developmental central nervous system deficiencies as well as ASD (Brimberg et al., 2013; Han et al., 2021). Another hypothesis relates increased gastrointestinal permeability, probably mediated by the immune system, with ASD onset (Navarro et al., 2015). This weakness of intestinal barrier might make ASD patients more vulnerable to environmental pathogens. Gluten and casein-derived molecules, if present in subjects with altered immune system, like in ASD, can cross the highly permeable intestinal barrier, and activate pro-inflammatory processes, releasing pro-inflammatory mediators that travel to the brain centers through the blood, contributing to neuro-inflammatory activities (De Magistris et al., 2013; Fiorentino et al., 2016).

## 5 Antioxidants targeting oxidative stress and neuroinflammation in ASD

Besides excessive levels of oxidative stress and pro-inflammatory molecules, ASD patients are also distinguished for their nutritional deficits. Several studies have pointed out that ASD subjects of all ages present nutritional abnormalities and redox imbalances caused by the dysfunctional mitochondria (Bjørklund et al., 2019). Therefore, adding antioxidants and anti-inflammatory molecules to the diet can significantly counterbalance their symptoms.

Several studies have proposed that ASD may involve different biological profiles, including alterations in redox balance, mitochondrial dysfunction and immune-inflammatory signaling. These profiles are supported by biochemical and metabolic findings, but they are not recognized as formal clinical subtypes, nor are they used to stratify participants in clinical trials. Consequently, the available evidence does not support direct matching between specific antioxidants and ASD subtypes. No clinical studies have evaluated antioxidant efficacy in biomarker-defined ASD groups, and standardized criteria for identifying such profiles remain lacking. However, since many antioxidants act on pathways that overlap with these observed biological alterations, we provide a brief summary of how the compound discussed in this review relates to the mechanisms most frequently implicated in ASD. This overview is intended to contextualize existing findings, without suggesting subtype-specific therapeutic applications (Table 1). Across compounds, the variability in dosing regimens, developmental timing, behavioral assays, and redox endpoints impose interpretive caution and require harmonization for future translational work. Finally, standardization of polyphenol preparations (e.g., percentage of trans-resveratrol, curcumin ≥95%) was inconsistently reported across studies, limiting cross-study comparability.

**TABLE 1:** Clinical summary of antioxidant intervention in ASD: Population, dose/route, duration, clinical domains, redox biomarkers, safety, and level of evidence are reported for each study.

Metabolite	Population (age/sex; setting)	Dose/Route	Duration	Primary clinical domains (effect)	Redox biomarkers (Y/N; which)	Safety/Tolerability	Level of evidence	Ref
Vitamin E	Children/adolescent with ASD (n = 17), ASD with co-morbid Intellectual disability (n = 19) Retrospective chart review study	Vitamin E (30-60 mg), Ubiquinol (50–100 mg), B-group vitamins	3 months	Improvements in cognition, adaptive functioning, social motivation, attention, and motor coordination; no placebo group	No (biomarkers not collected)	Radomized controlled necessary to confirm efficacy and tolerability	Low	Cucinotta et al. (2022)
Vitamin C (ascorbate)	Children with ASD (n = 24). DB-PC RTC	Glutathione (600 mg) intravenous, Vitamin C (2000 mg), N-acetylcysteine. Combined or glutathione alone	Two consecutive weeks, in a 8-week period, separated by a 1-week washout	No significant differences	Yes (quantification of reduced glutathione, oxidized glutathione and redox radius analyzed)	Generally well tolerated	Low	Williams et al. (2025)
Cysteine-rich whey protein isolate (CRWP), precursor to support Glutathione synthesis	Preschool Children with ASD 3–5 years (n = 46).; DB-PC RTC	Powder form: 0.5 g/kg for children<20 kg or 10 g for >20 kg children)	Daily doses for 90 days	Increased glutathione levels and selective improvements in adaptive behavior and socialization	Yes (quantification of reduced, oxidized, and total glutathione)	Generally well tolerated	Moderate-low	Castejon et al. (2021)
Resveratrol (add-on to risperidone)	Children with ASD 4–12 years (n = 62); DB-PC RCT	250 mg twice daily, oral	10 weeks	Hyperactivity -reduction; core ASD symptoms -not significant	No	Generally well tolerated	Low	Hendouei et al. (2020)
Coenzyme Q10 (ubiquinone-10)	Children 3–12 years with ASD (n = 90 of which boys = 66, girls = 24); randomized dosing comparison	30 or 60 mg/day, oral	100 days	Behavior: mixed signals; subgroup improvements reported	Yes MDA ↓; total antioxidant status (TAS)↑	Good overall tolerability	Low-Moderate	Mousavinejad et al. (2018)

Abbreviations: DB–PC, double-blind placebo-controlled; RTC, randomized clinical trial; FR-α Ab, folate receptor-alpha autoantibodies; TAS, total antioxidant status.

### 5.1 Vitamin E and ASD

Vitamin E is an antioxidant that, being part of the cellular membranes, interferes with or suppresses the lipid peroxidation while reducing reactive peroxyl radicals (Pangrazzi et al., 2020b). Moreover, vitamin E slows down the ROS production (Bej et al., 2024a). The study conducted by Langari employed a prenatal VPA model of ASD, in which pregnant rats received VPA on gestational days 8–10. Only male offspring were evaluated during adolescence. To test a potential protective effect, vitamin E (500 mg/kg) was administered prenatally 1 h before each VPA doses. Behavioral outcomes were assessed through the open field, three-chamber social interaction test and elevated plus maze, revealing improvements in repetitive behaviors, sociability, and anxiety-like response in vitamin E + VPA group (Alinaghi Langari et al., 2021).

Cucinotta et al., evaluated a metabolic antioxidant therapy combining ubiquinol Q10, Vitamin E, and B-complex vitamins in 59 individuals (42 males and 17 females) with neurodevelopmental disorders. In the cohort of 59 patients, 17 of them were diagnosed with ASD and 19 of them diagnosed with ASD co-occurring with intellectual disability or global developmental delay. Although the study provides the overall sex distribution, it does not specify the exact number of males and females within the ASD subgroup. Diagnoses were established according to DSM-V and confirmed through ADOS-2 or ADI-R. Patients received daily doses of ubiquinol (50–100 mg), vitamin E (30-60 mg), and B-group vitamins for at least 3 months. The study did not involve biological analyses; outcomes were evaluated through Clinical Global Impression ratings and clinician/parent reports. The intervention was associated with improvements in cognition, adaptive functioning, social motivation, attention, and motor coordination. The authors conclude that this antioxidant-based support is well tolerated and may provide clinically meaningful benefits, although controlled trials are needed to confirm efficacy (Cucinotta et al., 2022).

Weight of evidence: Overall evidence is *Low*: primarily preclinical with heterogeneous designs and no consistent human biomarkers.

### 5.2 Vitamin C and ASD

Vitamin C is a powerful antioxidant capable of counteracting and eliminating ROS produced during metabolic processes (Figueroa-Méndez and Rivas-Arancibia, 2015). In a study conducted by Qiu (Qiu et al., 2007) an increased vulnerability to oxidative damage in case of vitamin C deficiency was observed. Unlike vitamin E, vitamin C is not stored in the body and, since it is not endogenously produced, it is crucial that it is supplemented with a rich diet. When consumption is low or there is a malabsorption of vitamin C, scurvy, a rare disease may appear. This condition has also been observed in children with ASD, possibly resulting from highly selective and rigid eating behaviors (Kothari et al., 2020; Sakamornchai et al., 2022). Blood levels of vitamin C in those children are lower if compared to healthy controls (Marí-Bauset et al., 2015).

In a recent study on VPA-based rodent model, vitamin C was administered through drinking water (600 mg/L) from early gestation until the end on the third postnatal week, and only male offspring were evaluated. The treatment attenuated VPA-induced behavioral abnormalities in both Y-maze and social play tests, while also restoring prefrontal SOD and GSH-Px activity and normalizing VPA-related changes in neuronal number and soma size (Margedari et al., 2024).

Williams et al., studied the safety and preliminary efficacy of intravenous glutathione (600 mg), alone or combined with vitamin C (2000 mg) and N-acetylcysteine (up to 600 mg), in 24 children with ASD (glutathione group n = 6, 6 males and 0 females; trio group n = 6, 5 males and 1 female; placebo group n = 12, 10 males and 2 females), in a double blind, placebo controlled crossover design. The intervention was delivered as single weekly intravenous infusion, with each dose administered over 15 min in 50 mL of saline, for two consecutive 8-week periods, separated by a 1-week washout. Biological analyses were performed on plasma samples, quantifying total and reduced glutathione, oxidized glutathione and redox ratios using HPLC with colorimetric detection, while behavioral outcomes were assessed weekly through standardized caregiver questionnaires and clinician-rated scales. The study found no significant differences between active treatments and placebo in either biochemical or behavioral measures, although all groups showed mild improvements over time, likely reflecting nonspecific effects. The authors concluded that glutathione, with or without vitamin C and NAC, was well tolerated but did not produce measurable therapeutic benefit in this cohort (Williams et al., 2025).

Notably, no redox biomarkers were collected in this trial, which limits mechanistic interpretation.

Weight of evidence: Overall evidence is *Moderate–Low*: encouraging signal from a historical cross-over trial but limited by small samples and lack of biomarker data.

### 5.3 Glutathione and ASD

Glutathione is another important molecule, which ratio between the reduced form (GSH) and the oxidized one (GSSG) dictate and influences the redox status of the cell. It plays a very crucial role in supporting intracellular redox pathways, as it is one of the most significant antioxidants produced endogenously (Shen et al., 2000). Glutathione peroxidase (GPx) supports this system by catalyzing the reduction of hydrogen peroxide and lipid peroxides using GSH (Krishnamurthy et al., 2024). When the GSSG form is ten times as great to the GSH form, the cell is exposed to oxidative stress (Rushworth and Megson, 2014).

Notably, there is a correlation between neuronal apoptosis and GSH debilitation in the pathophysiology of ASD (Bjørklund et al., 2021). If the glutathione redox balance shifts towards the oxidized forms, it could serve as a precursor for DNA damage and possibly increased apoptosis, playing a crucial role as one of the causes of the disorder (Kulkarni and Wilson, 2008; Rose et al., 2008). Such results can also be supported by post-mortem findings of decreased GSH levels in blood, temporal cortex, and the cerebellum in ASD patients (Chauhan et al., 2012) while higher oxidized forms are present in ASD children (James et al., 2004). As reported, ROS generates hydrogen peroxide, which is a harmful molecule that can be scavenged by the glutathione peroxidase through GSH, it is found in the cytosol and the mitochondria, and by the catalase which is present in the peroxisomes. This main function of both enzymes can be threatened by the mitochondrial dysfunction mainly present in ASD children (Bjørklund et al., 2021). In autism, a reduction in glutathione levels is observed, but also a reduced methylation capacity and lower levels of sulfate, taurine and cysteine. Cysteine itself is an essential substrate for the synthesis of glutathione.

Evidence suggests that glutathione, when administered orally or transdermally, is inefficient in significantly raising systemic concentrations. Therefore, it has been suggested to provide precursors of glutathione (Kern et al., 2011). Moreover, supplementation of glutathione is not ideal because it is not transported inside the cells, indeed, it needs to be synthesized within them. A randomized, double-blind, placebo-controlled clinical trial conducted by Castejon et al., employed a cysteine-rich whey protein isolate (CRWP), a precursor designed to support glutathione synthesis. The trial enrolled 46 preschool children aged 3–5 years with a confirmed diagnosis of ASD, although the authors did not report the sex distribution, ASD subtypes, symptom severity, or comorbid conditions of the participants. Patients received CRWP (powder form: 0.5 g/kg for children<20 kg or 10 g for >20 kg children) daily for 90 days. Then behavioral outcomes were assessed, while molecular analyses quantified reduced, oxidized, and total glutathione in peripheral blood mononuclear cells (PBMCs). Children receiving CRWP showed a significant increase in glutathione levels and selective improvements in adaptive behavior and socialization domains (Castejon et al., 2021).

Glutathione is able to improve the antioxidative system decreasing the oxidative damage and the neuroinflammation caused to the neurons (Ghanizadeh et al., 2012). Interestingly, it has been shown that GSH levels tend to increase due to glutamate-mediated excitatory activity (Tebartz Van Elst et al., 2014), and one of the hypotheses related to symptoms of ASD is the presence of an imbalance between excitatory neurotransmitter, glutamate, and one of the principal inhibitory transmitter, GABA.

Weight of evidence: Overall evidence is *Moderate*: randomized data show improved cellular GSH with selective functional gains.

### 5.4 Quercetin and ASD

In addition to the compounds mentioned above, polyphenols also reveal promising and beneficial effects in ASD. Their main ability is to donate electrons to the oxidized molecules, being considered as powerful antioxidants (Ajami et al., 2017). Polyphenols can be helpful in improving cognitive function and neuroprotection (Castelli et al., 2018). From the family of polyphenols, the main one showing the most anti-inflammatory and antioxidant effects is the quercetin (Li et al., 2016). Quercetin (from *Quercus robur L* (Fagaceae), leaf; solvent not reported) is capable of enhancing mitochondrial activity while acting against oxidative stress and improving mitochondrial membrane potential (Bej et al., 2024b). The antioxidant properties of quercetin have been extensively characterized across different experimental models, including cardiotoxicity and heavy-metal-induced tissue injury. In a doxorubicin-induced cardiomyopathy model in male Sprague-Dawley rats Sharma et al., investigated the antioxidant and cytoprotective effects of quercetin. Cardiac tissue was examined for oxidative stress markers, including SOD, CAT, glutathione levels, and lipid peroxidation. Moreover, the authors performed q RT-PCR to quantify Nrf2 mRNA expression, demonstrating that quercetin upregulated this key antioxidant transcription factor while restoring endogenous antioxidant enzyme activity and reducing tissue damage (Sharma et al., 2020; Wang J. et al., 2020). Complementary evidence comes from Wang et al., who used cadmium-induced testicular toxicity model in male rats and showed that quercetin mitigated oxidative damage in the testes by evaluating SOD, GPx, CAT and GSH, lowering MDA levels, and reducing cadmium accumulation.

The potential neuroprotective effects of quercetin were also investigated by de Mattos et al., employing a prenatal VPA rat model of autism. Pregnant Wistar rats received quercetin at 50 mg/kg orally from gestational day 6.5–18.5, while autism-like features were induced by a single oral dose of VPA (800 mg/kg) on gestational day 12.5. The study evaluated both male and female offspring. Behavioral testing (open field, social interaction, tail flick nociception) was conducted between postnatal days 30–40, followed by biochemical analyses in cerebral cortex, hippocampus, striatum, and cerebellum. Oxidative stress was quantified through ROS, nitrites, TBARS, total thiols, and the activities of SOD, CAT GPx, GST, and ALA-D, using spectrophotometric assays and fluorescence-based detection. Quercetin prevented VPA-induced behavioral impairments and attenuated oxidative alterations across multiple brain regions, supporting its antioxidant and neuroprotective potential in this ASD model (De Mattos et al., 2020).

Since quercetin itself in low concentrations is poorly absorbed, can hardly cross the BBB and is quickly eliminated, the study of Moghaddam used quercetin-loaded nanophytosome (QNP) against autism-like damage in maternal separation model. Newborn Wistar rats (males only in the experimental phase were included) were separated from the dam for 3 h/day from postnatal day 1–9, and subsequently treated orally with quercetin or QNP at 10 or 40 mg/kg/day for 21 days starting at Post Natal Day (PND). Behavioral assessments (open-field, self-grooming, social interactions) were followed by biochemical and molecular analyses in the cerebellum, where oxidative stress markers (MDA, GSH, SOD, CAT, and GPx) were quantified spectrophotometrically, and Nrf2, Bax, Bcl-2, and Caspase-3 mRNA levels were measured by q RT-PCR. QNP showed superior efficacy to free quercetin, improving autism-like behaviors, restoring antioxidant enzyme activity, and modulating apoptotic and antioxidant gene expression (Moghaddam et al., 2023).

An additional consideration regarding quercetin concerns its structural classification as a pan-assay interference compound (PAINS). Although widely investigated for its antioxidant and bioactive properties, quercetin contains the catechol A substructure, which has been associated with assay interference and apparent promiscuity in high-throughput and biochemical screenings. This aspect highlights the need for careful interpretation of experimental findings, particularly when activity is reported (Bolz et al., 2021). In addition, brain-region specificity may influence antioxidant readouts, such as cortex, hippocampus, striatum, and cerebellum show distinct redox and metabolic profiles in ASD models.

Weight of evidence: Overall evidence is *Low* given potential assay-interference and PK constraints; clinical translation remains limited.

### 5.5 Resveratrol and ASD

Resveratrol (commonly sourced from *Vitis vinifera L* (Vitaceae), skin/seed or *Reynoutria japonica Houtt* (Polygonaceae), root), an important representative of the polyphenols, can stimulate mitochondrial activity inhibiting mitochondrial dysfunction which is typical of ASD patients (Jardim et al., 2018). This bioactive compound can protect cells from neuronal damage while reducing oxidative stress and improving memory (Moussa et al., 2017; Navarro-Cruz et al., 2017). The antioxidant activity of the resveratrol is exerted by its capacity to neutralize free radicals but also from its ability inactivating the antioxidant enzymes such as SOD, CAT, and GPx (Izzo et al., 2021). One of the main advantages of resveratrol is the fact that it can pass the BBB so it can be used as a targeting molecule in case of neurodegenerative diseases (Tellone et al., 2015). The administration of resveratrol in ASD patients enhances the social impairment and ameliorates the behavioral hyperactivity and cognitive function. The molecular mechanism through which resveratrol carries out this improvement in behavioral divergences is the expression of cortical gamma-amino butyric acid neurons, modulation of synthesis of anti-inflammatory molecules and antioxidants effects (Malaguarnera et al., 2020).

Resveratrol administration after prenatal exposure to VPA in rats, resulted in prevention or reduction of autism-like social deficits, pregnant Wistar females received a single intraperitoneal dose of VPA (600 mg/kg) on gestational day 12, while resveratrol was administered subcutaneously at 3.6 mg/kg/day from gestational day 6.5–18.5. Only male offspring were tested. Social behavior was assessed in adolescence using the three-chamber sociability and social novelty-test, without additional molecular or tissue-based analyses. Resveratrol (non-botanical resveratrol; trans-resveratrol ≥98–99%; botanical origin not reported) prevented VPA-induced deficits in sociability and social novelty preference, indicating a protective effect on autism-like social impairments (Bambini-Junior et al., 2014). Evidence suggests that the use of propionic acid (PPA) in rats might induce neuropathological changes similar to those observed in ASD.

In the study conducted by Bhandari and Kuhad, male Wistar rats received intracerebroventricular PPA infusions, followed by resveratrol treatment at 20 mg/kg/day for 10 days (non-botanical resveratrol; trans-resveratrol ≥98–99%; botanical origin not reported). Biochemical and histological analyses were performed on the hippocampal and cortical tissue, assessing oxidative stress markers, antioxidant enzyme activity, and glial activation through spectrophotometric assays and immunohistochemistry. Resveratrol significantly reduced lipid peroxidation, restored glutathione balance, and attenuated astrocyte and microglial reactivity, indicating a protective effect against PPA-induced oxidative and inflammatory damage (Bhandari and Kuhad, 2017). Differences in route (oral vs. subcutaneous), developmental timing, and brain-region sampling likely contribute to divergent findings across preclinical studies.

Zeng et al. (2024) employed a maternal immune activation (MIA) mouse model, induced by lipopolysaccharides (LPS) administration during pregnancy, which led to autism-like behaviors in the offspring. The authors hypothesized that resveratrol could mitigate these alterations by regulating Thoc5, a factor involved in RNA transport and processing. The downregulation of Thoc5 induced by LPS was restored in the offspring of resveratrol-treated mice and brought a significative reduction of pro-inflammatory cytokines in the placenta and fetal brain. The litter born from mothers administered with resveratrol, demonstrated a significative improvement in social interaction test and repetitive behaviors. These findings emphasize the critical role of the MIA in neurodevelopmental disorders and supports resveratrol’s protective potential (Zeng et al., 2024).

Some studies to assess the potential effects of resveratrol in ASD patients have also been conducted. In a double-blind, placebo-controlled randomized trial, Hendouei and colleagues evaluated resveratrol (non-botanical resveratrol; trans-resveratrol ≥98–99%; botanical origin not reported) as an adjunct to risperidone in children with ASD. Children aged 4–12 years were diagnosed with ASD according to DSM criteria, both boys and girls were included, and the study explicitly excluded participants with major psychiatric comorbidities such as ADHD, depressions, bipolar disorder, anxiety disorders, epilepsy, severe intellectual disabilities, or recent antipsychotic use, ensuring relative homogeneous sample focused on irritability. Patients (62 children) were randomized to receive risperidone plus resveratrol (250 mg twice daily) or risperidone plus placebo for 10 weeks. Clinical outcomes were assessed using Aberrant Behavior Checklist - Community (ABC-C) at baseline, week 5, and week 10, with no biological or imaging analyses performed. While resveratrol did not significantly improve the primary outcome of irritability, it produced a significant reduction in hyperactivity compared to placebo. Both boys and girls were included, but analyses were not sex-stratified (Hendouei et al., 2020).

Although often presented as a broadly beneficial polyphenol, resveratrol has also been repeatedly flagged as a potentially PAINS-susceptible molecule, owing to its redox activity, metal-chelating properties, and tendency to interfere with multiple assay formats in a non-specific manner as highlighted in the Nature commentary (Baell, 2016). Moreover, no redox biomarkers were collected in clinical trial, which limits mechanistic interpretation.

Weight of evidence: Overall evidence is *Low*: an add-on signal on hyperactivity without core-symptom change; no biomarker confirmation.

### 5.6 Curcumin and ASD

Curcumin (from *Curcuma longa L* (Zingiberaceae), rhizome; ethanolic extract, DER not reported; standardized to curcumin ≥95% when specified) exerts its major effects on reducing inflammation, oxidative stress and mitochondrial dysfunction (Bhandari and Kuhad, 2015). According to the study of Dong and collaborators, rats receiving curcumin manifest lower levels of pro-inflammatory cytokines such as IL-6 (Dong et al., 2012). This anti-inflammatory molecule can repress the pro-inflammatory gene expression while blocking the phosphorylation of the inhibitory factor I-kappa B (IkB).

In addition, curcumin is able to inhibit lipid peroxidation (El-Demerdash et al., 2009). It has been demonstrated that curcumin can act an important part in managing the protein aggregation, mitochondrial function and nitrosative/oxidative stress (Ak and Gülçin, 2008). Curcumin can cross the BBB improve the activity and increase the concentration of the antioxidant enzymes. These effects have been demonstrated *in vitro* in neuronal and glial models of oxidative stress, and *in vivo* in male Sprague-Dawley rats. These findings, although obtained from models of oxidative stress and mitochondrial dysfunction rather than in autism models, are highly relevant because they target biological mechanism that are also characteristic of ASD (Sachdeva et al., 2022).

Indeed, the study of Al-Askar et al., investigated the effect of curcumin (non-botanical source; standardized curcumin ≥95%.) in a VPA-induced rat model of autism. Pregnant Wistar rats received 600 mg/kg VPA on gestational day 12.5 and only male offspring were used. On PND 7, pups were treated orally with curcumin at 1 g/kg, and analyses were performed on whole-brain homogenates collected at day 28. The study quantified cytokines, neurotransmitters, oxidative stress markers and detoxification enzymes using ELISA-based assays, revealing that VPA exposure disrupted glutathione balance, increased IL-6, CYP450 and glutamate, and reduced serotonin and IFN-γ, while curcumin partially restored these alterations (Al-Askar et al., 2017).

Furthermore, Yang and Bhandari employed a PPA-induced rat model of autism, using male-only Sprague-Dawley rats that received intracerebroventricular infusions of 1M PPA. Curcumin (non-botanical source; standardized curcumin ≥95%) was administered orally for 28 days at 50, 100 or 200 mg/kg, and its effects were assessed through an extensive battery of neurobehavioral tests alongside biochemical analyses of whole-brain tissue. The study quantified oxidative and nitrosative stress markers, mitochondrial respiratory chain complex activities, and inflammatory mediators such as TNF-α and MMP-9, using spectrophotometric assays and ELISA. Curcumin dose-dependently reversed PPA-induced deficits in social interaction, repetitive behaviors, anxiety, locomotion, learning and memory, while normalizing oxidative stress, mitochondrial dysfunction and pro-inflammatory signaling, supporting its neuroprotective efficacy in this ASD model (Bhandari and Kuhad, 2015; Yang et al., 2020).

A noteworthy 2020 study employed the BTBR T^+^ tf/J (BTBR) mouse model of idiopathic autism to assess the effects of curcumin treatment from PND 6 to PND 8. Only male pups were used, and curcumin (non-botanical source) was administered intraperitoneally at 20 mg/kg/day. Behavioral testing at adulthood revealed improvements in sociability, reductions in repetitive behaviors and partial rescue of cognitive deficits. To uncover the underlying mechanisms, the researchers analyzed hippocampal tissue focusing on dentate gyrus, where they quantified neural progenitor proliferation and neurogenesis through BrdU, immunofluorescence for Sox2, GFAP, DCX and NeuN, and unbiased stereological cell counting. BTBR mice showed suppressed hippocampal neurogenesis, but curcumin restored progenitor proliferation and enhanced neural differentiation, suggesting that early curcumin exposure exerts long-lasting benefits (Zhong et al., 2020).

In another study on male BTBR mice curcumin demonstrated a modulatory effect on α7 nicotinic acetylcholine receptors (α7-nAChRs). It was administered intraperitoneally at 25, 50 or 100 mg/kg for 21 days, and behavioral analyses focused on sociability and repetitive behaviors. The beneficial effects were dose-dependent without altering locomotion or anxiety. Brain tissue from the hippocampus and cerebellum were examined. Using patch clamp electrophysiology, the researchers showed that curcumin potentiated α7-nAChRs in rat hippocampal neurons, and also restored SOD and CAT activity interactions (Jayaprakash et al., 2021).

Bioavailability remains a major translational challenge for oral curcumin, which undergoes rapid metabolism and shows limited systemic exposure unless delivered through optimized formulations (e.g., liposomes, nano-phytosomes).

Despite the promising therapeutic potential of polyphenols like curcumin and resveratrol, their clinical application is often hindered by poor bioavailability, rapid metabolism, and low chemical stability. However, recent advancements in novel drug delivery systems are overcoming these limitations. The development of nanotechnology-based formulations, such as liposomes and nanophytosomes, has shown great promise in enhancing the solubility, stability, and brain bioavailability of these compounds, thereby maximizing their neuroprotective effects in ASD (Moghaddam et al., 2023; Sachdeva et al., 2022).

However, it is important to note that curcumin has also been identified as a PAINS, exhibiting behaviors such as redox cycling, metal chelation, aggregation, and fluorescence interference that can artificially inflate its apparent bioactivity *in vitro* (Nelson et al., 2017).

Weight of evidence: Overall evidence is *Low–Moderate*: robust mechanistic plausibility and diverse preclinical benefits; clinical corroboration is needed.

### 5.7 Crocin, safranal and ASD

Crocin and safranal (*Crocus sativus L.* (Iridaceae), stigma; aqueous/ethanolic preparations; crocin/crocetin content reported when available) are both metabolites found in saffron (*Crocus sativus*). Safranal, a monoterpene aldehyde responsible for the characteristic smell of saffron, is an antioxidant that contributes to lowering oxidative stress in the neurons, inhibiting the production of pro-inflammatory cytokines and balancing the equilibrium between free radicals and antioxidants activity (Bej et al., 2024c). Crocin, on the other hand, is another metabolite of Saffron that also shows antioxidant activity that may be helpful in case of autism-like behavior since brain oxidative stress is compromised in the pathophysiology of the disease (Seyedinia et al., 2023).

The study of Seyedinia et al., was conducted on female rats that were injected with VPA on the 12.5th day of pregnancy to induce an experimental model of autism. The subsequent treatment of 28 days of saffron (30 mg/kg) or crocin (15 or 30 mg/kg) or saline was performed on the postnatal days 30–33 on male offspring. After 28 days of saffron treatment, behavioral testing targeting anxiety, nociception, motor coordination, and cognition showed an amelioration of autism-like behaviors. Moreover, biochemical analyses were performed on whole brain homogenates, assessing malondialdehyde, glutathione and catalase as oxidative stress markers using standard ELISA and spectrophotometric assays. The authors report that oxidative imbalance displayed by the offspring was counterbalanced (Seyedinia et al., 2023).

Another study reported that crocin administration (10, 30, or 50 mg/kg) for 28 days during the juvenile period in a prenatal VPA-induced autism model in rats, alleviated the autism-like behaviors in the male offspring. Behavioral assessments were conducted in the open-field test, while molecular analyses were performed on medial prefrontal cortex tissue. Gene expression on BDNF and SK-3 was quantified using real-time PCR, revealing that VPA reduced the expression BDNF, which plays a crucial role in synaptic plasticity and neuronal survival, and increased GSK-3, which is involved in neurodevelopment and neuroinflammation whereas crocin dose-dependently normalized both markers (Hosseini et al., 2025). The carotenoid profile of Crocus sativus L. (stigma) differs markedly from other botanical sources, potentially underpinning distinct redox signatures in VPA models.

Weight of evidence: Overall evidence is *Low–Moderate* in VPA models; clinical studies are pending.

### 5.8 L-carnosine, CoQ10 and ASD

Among the lipophilic nutraceuticals, Coenzyme Q10 (Q10) supplementation reduced markers of oxidative stress (Mousavinejad et al., 2018).

CoQ10 supplementation has a positive effect on oxidative stress in children with ASD; a randomized, placebo-controlled clinical study evaluated oral administration of Q10 in 90 children affected by ASD (66 boys and 24 girls), aged 3–12 years. They were all diagnosed according to DSM-IV-TR, ADOS, and CARS criteria; the study excluded individuals with comorbid neurological, metabolic, genetic or psychiatric conditions, including epilepsy, fragile X syndrome, tuberous sclerosis, PKU, fetal alcohol syndrome and any acute or chronic medical illness. Participants received 30 or 60 mg/day of Q10 for 100 days, and serum samples were analyzed for Q10 concentrations, malondialdehyde, total antioxidant status, SOD, and glutathione peroxidase, using ELISA assays. The study reported that the 60 mg/day dose significantly reduced lipid peroxidation and improved antioxidant status (Mousavinejad et al., 2018). CoQ10 bioavailability varies substantially by formulation, which may partly explain inconsistent behavioral findings across trials.

Furthermore, among the various nutraceuticals, L-carnosine stands out as a prominent compound believed to possess neuroprotective and antioxidant properties that may be beneficial for children with ASD. This molecule, combined with Q10 and α-lipoic acid, a mitochondrial coenzyme with potent antioxidant properties, was used to treat 16 children affected by autism (88% males). These children did not display any genetic syndromes, while only one child had a comorbid seizure disorder. All children showed mitochondrial enzymatic abnormalities on the MitoSwab assay at baseline. Biological analyses were performed on PBMCs, assessing mitochondrial enzymology (citrate synthase and ETC complexes) and mitochondrial respiration using Seahorse XFe96 with the mitochondrial oxidative stress test. The supplement normalized citrate synthase and complex IV activity and enhanced mitochondrial resilience to oxidative stress, particularly in children with greater developmental impairment (Hill et al., 2025). Reported clinical heterogeneity may, in part, reflect formulation-dependent variability in CoQ10 bioavailability.

Weight of evidence: Overall evidence is *Moderate*: pediatric trials show lipid-peroxidation reduction and improved antioxidant status, with mixed behavioral effects.

## 6 Indirect antioxidants

While some molecules act like antioxidants by directly scavenging free radicals, there are some bioactive compounds that can support oxidative stress balance indirectly, by regulating inflammatory pathways, redox signaling or mitochondrial function. Notably, polyunsaturated fatty acids (PUFAs), folates and probiotics have shown positive effects on these aspects (Table 2).

**TABLE 2:** Clinical summary of indirect antioxidant intervention in ASD.

Supplementation	Population (age/sex; setting)	Dose/Route	Duration	Primary clinical domains (effect)	Redox biomarkers (Y/N; which)	Safety/Tolerability	Level of evidence	Ref
Omega-3	Children with ASD (n = 54; control group 20 males and 6 females; intervention group 19 males and 9 females); RTC	1,000 mg/day of long-chain omega 3 (189 mg EPA +120 mg DHA) or placebo; oral	8 weeks	Improved stereotyped behaviors, social communication, and overall GARS scores compared to placebo	No (biomarkers not collected)	Well tolerated	Low	Doaei et al. (2021)
Omega 3-6-9	Children 18–38 months, who were either born preterm or exhibited early signs of ASD (31 participants, 68% males); DB RTC	Omega 3-6-9 (338 mg EPA, 225 mg DHA, 93 mg GLA, 306 mg oleic acid per day) or placebo; oral	Daily for 90 days	Medium improvements in anxious/depressed behaviors and internalizing symptoms, and a large improvement in interpersonal relationship skills, but no effects on sleep or other behavioral domains	No (biomarkers not collected)	Well tolerated	Low	Boone et al. (2022)
Fish oil	Young adults 19–40 years (19 participants, 15 males and 4 females) with moderate-to-profound intellectual disability; Open Label study	Fish oil capsules weeks (0.93 g EPA + DHA) plus 5 mg vitamin E	Two capsule/day for 6 weeks	The intervention produced no significant improvement in severity or frequency of aberrant behaviors during treatment or follow-up	No (biomarkers not collected)	Well tolerated	Low	Politi et al. (2008)
Efalex®	Children with ASD 3–11 years (30 participants; 18 males and 12 females) and 30 healthy children; Non-randomized, uncontrolled pre–post intervention study	DHA (60 mg), EPA (13 mg), gamma-linolenic acid (GLA) (12 mg) and arachidonic acid (AA) (5 mg)	Two capsules twice a day for 3 months	After treatment, PUFA concentration increased significantly, particularly linoleic acid and DHA, and 20 of 30 children showed measurable behavioral improvement	No (biomarkers not collected)	Well tolerated	Low	Meguid et al. (2008)
Folinic acid (calcium folinate)	Children with ASD and language impairment (48 participants; 82% males and 18% females) DB-PC RCTs (FR-α Ab stratification)	1–2 mg/kg/day (max 50 mg/day), oral	12–24 weeks	Language/communication improvement; stronger in FR-α autoantibody-positive subgroup	Variable (often not primary endpoints)	Good; no major safety signals reported	Moderate (Higher in FR-α Ab + endophenotype)	Frye et al. (2018)

Abbreviations: DB–PC, double-blind placebo-controlled; RTC, randomized clinical trial; FR-α Ab, folate receptor-alpha autoantibodies; TAS, total antioxidant status.

### 6.1 PUFAs and ASD

Among the PUFAs, the omega-3 and omega-6 series are the most common (Veselinović et al., 2021), their presence is vital for proper neurological maturation and activity (Doaei et al., 2021).

Recent findings suggest that omega-3 fatty acid, such as EPA and DHA, may act as indirect antioxidants in ASD. These compounds act through modulation of neuroinflammation and oxidative stress by regulating microglial activation and reducing proinflammatory cytokines (Veselinović et al., 2021).

A preclinical study by Alfawaz et al., employed a propionic acid (PPA)–based rat model. The experimental design included 35 juvenile male Western albino rats, distributed into five groups of seven animals each: untreated controls, PPA alone, and PPA followed by treatment with omega-3, vitamin B12, or their combination. The researchers examined the impact of these interventions through a biochemical characterization of brain homogenates, quantifying oxidative and inflammatory markers such as lipid peroxides, glutathione (GSH), glutathione S-transferase (GST), 5-lipoxygenase (5-LOX), cyclooxygenase-2 (COX-2), leukotriene B4, prostaglandin E2, and ascorbic acid using spectrophotometric assays and ELISA. They also assessed gut microbial composition by culturing fecal samples on selective media. PPA exposure produced pronounced oxidative stress and disruptions in lipid metabolism, whereas post-treatment with omega-3 or vitamin B12, administered individually or together, partially normalized antioxidant defenses, modulated inflammatory enzyme activity, and altered microbial profiles. These findings indicate that omega-3 and vitamin B12 can counteract several PPA-induced biochemical abnormalities, although the evidence remains limited to an animal model and cannot be directly extrapolated to clinical populations (Alfawaz et al., 2018).

The study of Doaei evaluated whether omega-3 supplementation modifies behavioral and social symptoms in children diagnosed with ASD according to DSM IV-TR criteria and ADOS assessment. 54 participants (control group: 20 males and 6 females, intervention group: 19 males and 9 females. The intervention consisted of 1,000 mg/day of long-chain omega-3 (189 mg EPA +120 mg DHA) for 8 weeks, compared with a placebo containing medium chain triglycerides. Behavioral outcomes were assessed using Gilliam Autism Rating Scale-2, while dietary intake was quantified through a validated food-frequency questionnaire. Omega-3 supplementation significantly improved stereotyped behaviors, social communication, and overall GARS scores compared to placebo (Doaei et al., 2021).

Furthermore, in a study involving 31 children aged 18–38 months who were either born preterm or exhibited early signs of ASD, participants received omega-3-6-9 supplementation (338 mg EPA, 225 mg DHA, 93 mg GLA, 306 mg oleic acid per day) or placebo. The sample included both males and females (68% boys), children with major neurological syndromes, severe developmental delay, sensory impairments, or medical conditions were excluded. Outcomes relied on caregiver-reported behavioral and sleep questionnaires (CBCL, TBAQ-SF, Vineland-2, CSHQ, BISQ), analyzed using mixing effects regression models. Supplementation produced medium improvements in anxious/depressed behaviors and internalizing symptoms, and a large improvement in interpersonal relationship skills, but no effects on sleep or other behavioral domains (Boone et al., 2022).

The study by Politi et al., enrolled 19 young adults aged 18–40 (15 males and 4 females) diagnosed according to DSM-IV-TR and CARS scores>40, all presenting moderate-to-profound intellectual disability. Participants received two fish-oil capsules daily for 6 weeks (0.93 g EPA + DHA) plus 5 mg vitamin E. Outcomes relied exclusively on caregiver-rated behavioral assessments using Rossago Behavioral Checklist. The intervention produced no significant improvement in severity or frequency of aberrant behaviors during treatment or follow-up (Politi et al., 2008).

The study of Meguid et al., involved 30 healthy children and 30 children affected by autism (18 males, 12 females), aged 3–11 years, all diagnosed with DSM-V and CARS, with no reported medical comorbidities and in good physical health. Children received Efalex®, a fish oil-based supplement containing DHA (60 mg), EPA (13 mg), gamma-linolenic acid (GLA) (12 mg) and arachidonic acid (AA) (5 mg), two capsules twice daily for 3 months. Biochemical analyses were performed on dried whole-blood spots, assessing free PUFAs using tandem mass spectrometry, while behavioral changes were evaluated with CARS. Before supplementation, ASDchildren showed markedly reduced levels of linoleic acid, DHA, AA and linoleic acid compared with healthy controls (Meguid et al., 2008). Notably, no redox biomarkers were collected in this trial, which limits mechanistic interpretation. Weight of evidence: Overall evidence is *Low,* some RCTs document benefits for social communication and internalizing symptoms, but findings are inconsistent across age groups and severity levels, and no study integrates redox biomarkers to confirm mechanistic effects.

### 6.2 Folates and ASD

Folic acid, folinic acid and 5-methylenetetrahydrofolate are folates, which are essential for the metabolism of nucleotides and DNA methylation. Folate metabolism plays an essential role in maintaining redox homeostasis in ASD by regulating homocysteine levels and supporting glutathione synthesis, key components in defense against oxidative stress. Indeed, Rossignol and Frye (Rossignol and Frye, 2021) examined studies linking cerebral folate deficiency to ASD. Treatment studies administering folinic acid at doses typically ranging from 0.5 to 2 mg/kg/day reported improvements in communication, irritability, attention, stereotypy, and neurological signs. Many studies have questioned if an increase in maternal folate during pregnancy, with the main aim of reducing neural defects, could somehow be related to an increased risk of ASD in children because the timing of neural tube closure, and therefore the use of folic acid corresponds with embryogenesis. A comprehensive review of recent clinical data highlights that while folate deficiency is common among individuals with ASD, maternal folic acid supplementation does not correlate with higher risk of the disorder, indeed folate therapy has been associated with improved neurodevelopmental outcomes (Vasconcelos et al., 2025).

Frye and collaborators conducted a study on 48 non-syndromic ASD children with language impairment, diagnosed using ADOS, ADI-R or multidisciplinary clinical criteria. The cohort included 82% males and 18% females, and children with genetic syndromes, prematurity, major medical disease or severe irritability were excluded, although common comorbidities such as gastrointestinal, allergic, neurological, and sleep disorders were present. Biological analyses were performed on fasting blood samples, measuring folates, B12, zinc, cooper, magnesium, glutathione redox status, and folate receptor-alpha autoantibodies using immunoassays and biochemical assays. Folinic acid (2 mg/kg/day, max 50 mg/day) produced significantly greater improvements in standardized verbal communication scores and several adaptive and behavioral subscales compared with placebo, with a stronger effect on children (Frye et al., 2018).

Furthermore, a placebo-controlled randomized trial evaluated the efficacy of folinic acid (5 mg twice daily) for 12 weeks in 19 children aged 3–10 years (sex distribution not reported) with ASD diagnosed by ADOS, without genetic syndromes, major medical comorbidities, or treatments affecting folate metabolism. Biological analyses were performed on serum, measuring folate, vitamin B12, homocysteine, and folate receptor-alpha autoantibodies using radioimmunoassay and mass spectrometry. Folinic acid produced significant improvements in global ADOS score, social interaction, and communication, whereas placebo showed no meaningful change (Renard et al., 2020).

Ramaekers et al., observed lower levels of 5-MTFH, the active form of folic acid, in the cerebrospinal fluid (CSF) in ASD patients. This was due to the presence of autoantibodies against folate receptor which blocked the folate binding site. Indeed, in 23 patients out of a total of 25, 5-MTHF in CSF was low despite normal serum folate treatment with folinic acid for a year restored folate levels and led to clinical improvements. Neggers et al., confirmed these observations, highlighting that over 70% of children affected by ASD showed positivity to these autoantibodies and may benefit from targeted folinic acid supplementation (Neggers, 2014; Ramaekers et al., 2007; Ramaekers et al., 2013). The presence of folate receptor-α autoantibodies represents a biologically defined ASD endophenotype with higher responsiveness to folinic acid, illustrating the value of biomarker-guided intervention.

Weight of evidence: Overall evidence is *Moderate–High* in FR-α Ab + children; smaller or inconsistent effects outside this endophenotype.

### 6.3 Prebiotics, probiotics and ASD

Recent findings suggest that prebiotics and probiotic supplementation may counteract oxidative stress in individuals with ASD, likely through modulation of microbial composition and lowering oxidative stress biomarkers.

Indeed, a study employed a PPA rodent model of autism to examine whether probiotic supplementation can counteract gut barrier dysfunction and neurochemical alterations. 40 juvenile male Western Albino rats (aged 3–4Ramaekers et al., weeks) received oral PPA at 250 mg/kg/for 3 days to induce autism-like features, followed by 3 weeks of probiotic treatment with *Bifidobacterium infantis*, *Lactobacillus bulgaricus,* or a commercial probiotic mixture (all at 0.2 g/kg). Brain tissue homogenates and serum samples were collected to quantify intestinal permeability markers (zonulin and occluding), oxidative-stress markers (GSH, MDA, CAT), and GABA-receptor gene expression using ELISA and biochemical assays. The study concluded that probiotics reduced gut leakiness, lowered oxidative stress, and enhanced GABA-receptor expression, supporting a protective role on the gut-brain axis in this ASD model. Among the tested interventions, *Bifidobacterium infantis* produced the most pronounced effects, showing the greatest reduction in gut-leakiness markers and the strongest upregulation of GABA-receptor expression (Bin-Khattaf et al., 2024). Also, according to Zarezadeh et al., the restrictive dietary behaviors in ASD may exacerbate oxidative stress within the gut, proposing that targeted probiotics therapies may reduce markers of lipid peroxidation and enhance oxidative stress defenses (Zarezadeh et al., 2023).

Moreover, Wang et al., used a maternal immune activation (MIA) mouse model to examine whether probiotic supplementation during pregnancy can prevent autism-related phenotypes in offspring. Pregnant C57BL/6J dams received Poly (I:C) at 20 mg/kg on embryonic day 12.5, while a probiotic mixture containing *Bifidobacterium bifidum, B. infantis* and *Lactobacillus helveticus* was administered orally from embryonic day 0.1 to PND21, only male offspring were tested. Behavioral assessment included a comprehensive battery: three-chamber social interaction, social novelty, marble burying, self-grooming, open-field, elevated plus maze, tail suspension, and forced-swim test. Biochemical analyses were performed on maternal serum, fetal brains, and adult prefrontal cortex, measuring IL-6 and IL-17a (ELISA), parvalbumin-positive interneurons (immunofluorescence), and GABA/glutamate levels (HPLC). Probiotic treatment prevented MIA-induced increases in IL-6/IL-17a, preserved parvalbumin positive interneurons, normalized GABAergic signaling, and fully rescued social, repetitive, anxiety-and depression-like behaviors in adult offspring (Wang et al., 2019).

Another study investigated whether prebiotic and probiotic exert neuroprotective and antioxidant effects in a PPA rat model of ASD. The study used 36 male Sprague-Dawley rat pup, exposed to 250 mg/kg PPA to induce ASD-lke features, and pre-treated for 27 days with either yogurt, artichoke extract (400 mg/kg), luteolin (50 mg/kg) or *Lacticaseibacillus rhamnosus GG* (0.2 mL/day). Biochemical analyses were performed on brain tissue homogenates, quantifying GSH, GPX1, GABA, IL-6, IL-10 and TNFα using ELISA-based assays. PPA exposure markedly increased oxidative stress and neuroinflammation, while all nutritional intervention partially restored antioxidant markers and reduced pro-inflammatory cytokines, with *L. rhamnosus GG* showing the strongest GPX1 recovery. The authors concluded that probiotic and prebiotic supplementation exerts measurable antioxidant and anti-inflammatory effects in this model, supporting their potential as complementary neuroprotective strategies (Alsubaiei et al., 2023).

Supplementation with three different *lactobacillus* strains to VPA-induced ASD mice improved behavioral symptoms compared to the risperidone group, a drug approved by the Food and Drug Administration. In this study, only male offspring were included, from weaning to sexual maturation (4–8 weeks of age), rats were treated by gavage with *Lactobacillus helveticus CCFM1076, L. acidophilus La28, or L. acidophilus JCM1132* at 10^9^ CFU/mL. Behavioral testing was followed by biochemical analyses on colon, faces, serum, cerebellum, and prefrontal cortex, assessing tryptophan metabolites, serotonin (5-HT) pathway intermediates, neurotransmitters (GABA, glutamate, dopamine, acetylcholine) via UPLC, UPLC-TQD, and ELISA, alongside gut microbiota profiling through 16S r RNA sequencing. Among the tested strains, *Lactobacillus helveticus CCFM1076* showed the strongest therapeutic effect, improving social behavior, restoring 5-HT synthesis and metabolism in both peripheral and central tissues, balancing excitatory-inhibitory neurotransmission, increasing hypothalamic oxytocin, and partially normalizing gut dysbiosis (Kong Q. et al., 2021).

The mechanism by which probiotics exert their positive effects is still not known but it is though that they might influence neuroactive metabolites like gamma-aminobutyric acid and serotonin (Israelyan and Margolis, 2018). Most of these findings come from animal models, where probiotics have been shown to influence GABAergic signaling (e.g., altered expression of GABA_A and GABA_B receptor subunits in the brain) and serotoninergic pathways (such as changes in tryptophan metabolism, enterochromaffin-cell 5HT synthesis, and SERT-mediated reuptake), highlighting potential mechanisms through which the gut microbiota may affect brain function (Israelyan and Margolis, 2018). When probiotics are combined with fructooligosaccharides, a reduction in the hyper-serotonergic state is observed (Wang Y. et al., 2020).

According to Grimaldi and his research group, the use of prebiotics in individuals with ASD has been shown to improve levels of the beneficial bacteria, inhibiting harmful bacteria and reducing the anti-social behavior present in many patients (Grimaldi et al., 2018; Wang Y. et al., 2020). They evaluated the effects of a 6-week prebiotic intervention with B-GOS® galactooligosaccharides in 30 children diagnosed with ASD. The cohort included both males and females although the exact sex distribution is not reported, and children with major medical comorbidities were not described beyond gastrointestinal complaints. Participants either followed an exclusion diet, defined as gluten and casein free regimen adopted independently by families before enrolment, or an unrestricted die, an important source of baseline heterogeneity, as dietary patterns were not experimentally assigned. The study analyzed fecal and urine samples to assess gut microbiota composition (via 16S rRNA sequencing and FISH) and metabolic profiles (via ^1^H-NMR spectroscopy), with behavioral questionnaires and GI symptom diaries. Result showed that children on exclusion diets were associated with distinct microbial and metabolic profile at baseline, and that B-GOS® improved social behavior only in children already following the exclusion diet, while also inducing specific shift in gut microbiota and metabolites. However, because dietary groups differed prior to intervention, the study cannot fully disentangle prebiotic effects from those attributable to the pre-existing diet.

According to the study of Shaaban et al., if ASD children aged 5–9 years are given a supplementation a 3 months supplementation with *Lactobacillus acidophilus*, *Lactobacillus rhamnosus* and *Bifidobacterium longum,* significant improvements emerge in ASD severity, gastrointestinal symptoms, and domains related to social interaction and communication. The study enrolled 30 children with ASD (19 males and 11 females) diagnosed according to DMS-V and confirmed through ADOS/ADI-R, while excluding those with neurodevelopmental comorbidities, chronic medical conditions, gastrointestinal diseases, or special diets. Participants received 5 g/day of probiotic powder (100 × 10^6^ CFU/g), and stool samples were analyzed by quantitative real time PCR to quantify *Lactobacillus rhamnosus* and *B. longum.* Behavioral and gastrointestinal outcomes were assessed using ATEC and 6-item GI Severity Index, both of which showed marked improvement alongside increased counts of beneficial gut bacteria. Although promising, the absence of blinding and placebo control limits the strength of causal inference (Shaaban et al., 2018).

The study by Billeci et al., demonstrated that the use of probiotics for 6 months can modify the electroencephalography patterns, showing reduction in β and γ bands power in frontopolar regions, frequency ranges often implicated in higher-order cognitive processing, and increases in coherence and frontal asymmetry toward patterns more consistent with neurotypical development. These neurophysiological shifts were observed in preschoolers with ASD (35 males and 11 females) diagnosed through to DSM-5 and validated with ADOS-2 and ADI-R, with participants screened to exclude neurological syndromes, epilepsy, sensory impairments, gastrointestinal diseases, secial diets, and other major comorbidities. Children received a high-dose multistrain probiotic mixture (De Simone Formulation, 450 billion CFU/day). These findings suggest that probiotics may modulate neurophysiological activity in ASD (Billeci et al., 2023).

A double-blind, placebo-controlled pilot trial, evaluated the effects of *Lactobacillus plantarum PS128* and intranasal oxytocin in 35 individuals with ASD (26 males, 9 females) aged 3–20 years. All participants had a diagnosis of ASD established with DSM-IV-TR or DSM-5 and validated with ADOS-2 and/or ADI-R, while individuals with neurological or psychiatric comorbidities, recent probiotic or oxytocin use, or unstable medications were excluded. Subjects received the probiotic at 6 × 10^6^ CFU/day for 16 weeks, followed by an additional 12 weeks of combined treatment with intranasal oxytocin titrated up to 32 IU/day. Biological analyses were performed on stool samples (16S rRNA sequencing) and blood serum (ELISA for oxytocin, inflammatory and neuroinjury markers), while behavioral outcomes were assessed using SRS, ABC and CGI. The combination of probiotics and oxytocin produced greater improvements in social responsiveness, aberrant behaviors, and clinician-rated global functioning than either treatment alone, and was associated with favorable shift in gut microbiome network structure. The authors concluded that *Lactobacillus plantarum PS128* and oxytocin may exert synergistic effects on socio-behavioral symptoms in ASD, warranting larger controlled trials (Kong X.-J. et al., 2021).

Many of the studies reviewed highlight promising behavioral, neurochemical, or microbiota-related effects of probiotic and prebiotic interventions, yet very few include direct measurements of oxidative-stress biomarkers. As a result, evidence for antioxidant action in humans remains largely indirect and inferred from animal models, underscoring the need for clinical trials that systematically quantify redox outcomes alongside behavioral endpoints.

Weight of evidence: Overall evidence is *Low-Moderate:* multiple animal studies and a growing number of small clinical trials suggest benefits across gut–brain signaling, social and behavioral domains, gastrointestinal symptoms, and neurophysiological patterns, yet most human studies rely on caregiver-reported outcomes, lack biomarker integration, and seldom control for diet, comorbidities, or baseline microbiome composition. Evidence for antioxidant action remains largely indirect, without systematic measurement of redox biomarkers. Larger, placebo-controlled, biomarker-guided trials are needed to clarify efficacy, mechanisms, and optimal microbial strains.

Taken together, these heterogeneous findings highlight both biological plausibility and the methodological constraints that shape the current evidence base, motivating the integrative Discussion below.

## 7 Discussions

A growing body of evidence indicates that oxidative stress, mitochondrial dysfunction, and neuroinflammation are central to the pathophysiology of ASD. These mechanisms affect synaptic plasticity, energy metabolism, and the integrity of the gut–brain barrier. Postmortem and imaging studies have revealed electron transport chain defects and impaired pyruvate dehydrogenase activity in ASD brains, with regional reductions in Complex I availability and non-ETC enzymes such as aconitase and PDH. These findings support a causal role of mitochondrial bioenergetics in neurobehavioral phenotypes (Chauhan et al., 2011; Tang et al., 2013; Gu et al., 2013; Kato et al., 2023). Complementary clinical and meta-analytic data consistently demonstrate systemic redox imbalance, including lower glutathione reserves, altered GSH/GSSG ratios, increased lipid peroxidation, and dysregulated NRF2 signaling, linking oxidative status to immune activation and synaptic dysfunction (James et al., 2004; Chauhan et al., 2012; Chen et al., 2021; Davinelli et al., 2025). These mechanisms plausibly influence behavioral domains (irritability, hyperactivity, sociability, language, sleep) and interact with gastrointestinal comorbidities and barrier integrity.

Within this mechanistic framework, natural compounds with antioxidant and anti-inflammatory properties, including vitamins C and E, glutathione precursors, CoQ10, L-carnosine, polyphenols including quercetin, resveratrol, and curcumin, and saffron metabolites like crocin and safranal show biological plausibility and preliminary efficacy on biochemical and behavioral endpoints. Clinical studies, although limited in size, report improvements in irritability, hyperactivity, sleep quality, communication, and oxidative stress biomarkers following supplementation with vitamin C, CoQ10, folinic acid, and mitochondrial support formulations (Mousavinejad et al., 2018; Frye et al., 2018; Renard et al., 2020; Hill et al., 2025). Preclinical models demonstrate that these compounds reduce reactive oxygen species, stabilize mitochondrial membrane potential, enhance mitophagy, and activate NRF2 dependent transcription, which translates into attenuation of ASD like behaviors in VPA, propionic acid, maternal immune activation, and BTBR paradigms (Bambini Junior et al., 2014; De Mattos et al., 2020; Yang et al., 2020; Zhong et al., 2020; Zeng et al., 2024). Advances in drug delivery systems, including liposomes and nanophytosomes, address low solubility, rapid metabolism, and limited blood brain barrier penetration, thereby improving central nervous system exposure and pharmacodynamic effects, which is essential for clinical translation of several phytochemicals (Moghaddam et al., 2023; Sachdeva et al., 2022).

Indirect antioxidants and systems level modulators such as PUFAs, folates or folinic acid, and prebiotics or probiotics also show promise. By influencing membrane composition, immune signaling, and gut microbiota, these interventions have been associated with improvements in social motivation, adaptive behavior, anxiety, and gastrointestinal symptoms, together with reductions in lipid peroxidation markers and enhanced barrier function (Veselinović et al., 2021; Santocchi et al., 2020; Bin Khattaf et al., 2024; Zarezadeh et al., 2023). This evidence supports a multi target therapeutic paradigm that is consistent with the multifactorial nature of ASD and suggests that combined interventions, for example, omega3 with vitamin D or vitamin E, or cysteine rich proteins to boost glutathione, may exert additive or synergistic effects across redox, mitochondrial, and immune pathways (Castejon et al., 2021; Politi et al., 2008; Boone et al., 2022). This mapping is hypothesis-generating and should not be construed as subtype-specific therapeutic guidance until biomarker-stratified trials are conducted.

Despite these promising signals, important limitations constrain interpretation and translation. A source of inconsistency arises from preclinical studies, which differ markedly in their methodological approaches. Animal models employ a wide range of behavioral assays, biochemical endpoints, and tissue sampling strategies, making cross-study comparison inherently difficult. Some investigators analyze whole-brain homogenates, whereas others focus on specific regions such as cortex, hippocampus, or cerebellum, each characterized by distinct redox and metabolic profiles. Moreover, the choice of behavioral tests varies substantially across studies, which further complicates the interpretation of antioxidant effects. This methodological heterogeneity mirrors the variability observed in human studies. Indeed, the clinical literature exhibits heterogeneity in design, dosing, duration, and outcome measures, frequent reliance on parent reported scales without uniform blinding, and underpowered samples that hinder strong inference and guideline development (Doherty et al., 2022; Vasconcelos et al., 2025). Few studies stratify participants by biological signatures such as mitochondrial phenotypes, redox profiles, or folate receptor alpha autoantibodies, even though data indicate differential responsiveness to targeted interventions in defined endophenotypes (Frye et al., 2024; Rossignol and Frye, 2021). The current literature does not support a direct association between specific antioxidants and clinically defined ASD subtypes. Pediatric pharmacokinetics and long-term safety require clarification, particularly for polyphenols and carotenoids with intrinsically low oral bioavailability and potential interactions with conventional medications like antipsychotics or stimulants (Hendouei et al., 2020; Mousavinejad et al., 2018). In addition, both preclinical and clinical literature often underrepresent females, limiting insights into sex-specific immunometabolic and hormonal modulators of treatment response (Seyedinia et al., 2023; De Mattos et al., 2020). These mechanisms have not been systematically investigated in ASD populations, and no antioxidant trials to date have reported sex-specific treatment outcomes. A further methodological issue is that many *in vivo* studies explicitly exclude female animals to avoid potential confounding effects related to hormonal fluctuations. Although this practice is common across biomedical research, it has unintended consequence of systematically removing female-specific biological variability from the evidence base. As a result, our understanding of redox regulation, mitochondrial function and neuroimmune signaling in ASD females remain extremely limited. This gap is particularly problematic given that ASD in females often presents with distinct behavioral, developmental, and physiological profiles, which may influence both oxidative stress-markers and response to antioxidant-based interventions. Incorporating sex as a biological variable in future studies will therefore be essential to determine whether males and females may benefit differently from antioxidant-based treatments and to address the current imbalance in ASD research.

It is also important to note that many ASD studies systematically exclude participants with common comorbidities such as epilepsy, gastrointestinal disorders or severe behavioral dysregulation. The aim is to minimize confounding factors and obtain cleaner biological signals. However, this practice inevitably narrows the clinical spectrum represented in research samples and reduces natural heterogeneity of ASD, limiting the generalizability of findings. This issue is clearly illustrated by Politi et al., who investigated omega-3 supplementation in a cohort of young adults with severe autism and profound intellectual disability, reporting no behavioral benefits. In contrast, several studies included in this review that enrolled younger individuals or participants with milder symptom profile have documented improvements following EPA/DHA supplementation, suggesting that age, developmental stage, and baseline severity may critically modulate treatment responsiveness.

Another recurring limitation emerges on antioxidant-based interventions in ASD, constrained by a fundamental methodological gap: most clinical studies report behavioral or functional improvements without directly measuring oxidative-stress biomarkers. This disconnect limits the ability to determine whether observed benefits truly reflect restoration of redox homeostasis or arise from unrelated mechanisms. As a result, the antioxidant hypothesis ASD remains supported largely by preclinical evidence and by indirect clinical observations rather than by robust biochemical validation in patients.

To address these gaps, future research should prioritize larger scale multicenter randomized controlled trials with standardized protocols, adequately powered biomarker-guided RCTs with harmonised clinical endpoints plus GSH/GSSG, MDA/TBARS, SOD/CAT/GPx panels, head-to-head monotherapy vs. combination designs, sex-stratified analyses, PK-informed formulations for polyphenols, and developmentally sensitive timing. Precision medicine approaches should enroll participants based on biological signatures rather than broad diagnostic categories, including glutathione status, lactate to pyruvate ratios, N acetyl aspartate, NRF2 related profiles, electron transport chain complex activity, and the presence of folate receptor alpha autoantibodies in cerebral folate deficiency. Development and testing of advanced delivery strategies, such as nanoformulations and prodrugs, are needed to overcome bioavailability challenges and improve central nervous system penetration and target engagement. Ethnopharmacological knowledge can guide the design of synergistic phytochemical combinations that move beyond single compound interventions toward standardized multi component formulations with complementary actions on redox balance, membrane biology, and immune modulation (Bej et al., 2024c; Yang et al., 2020). Trials should also incorporate sex-specific analyses, age related stratification, and clinically relevant comorbidities such as gastrointestinal dysfunction and sleep problems, all of which can modulate treatment efficacy. Finally, establishing pharmacovigilance frameworks and core outcome sets with clinically meaningful thresholds will be essential to ensure safety, comparability, and regulatory readiness for antioxidant-based strategies in ASD.

Overall, current data substantiates a strong mechanistic rationale and an early clinical signal for natural compounds in ASD, especially in subgroups with oxidative or mitochondrial dysfunction or nutritional deficits. With rigorous methodology, biomarker guided enrollment, optimized delivery, and careful safety monitoring, antioxidant-oriented interventions can progress from complementary approaches to validated therapeutic options that complement existing standards of care for defined ASD endophenotypes.

## 8 Conclusion

To conclude, ASD is a multifaceted neurodevelopmental condition shaped by a combination of genetic, environmental, and immune-related influences. Oxidative stress and neuroinflammation are increasingly recognized as central elements in its development, with mitochondrial dysfunction and weakened antioxidant systems playing a significant role. Natural compounds with antioxidant and anti-inflammatory properties offer biologically plausible and clinically promising strategies, particularly for subgroups with redox imbalance or nutritional deficiencies. Although compounds such as vitamins, polyphenols, and plant-derived molecules have demonstrated promise in preliminary studies, more comprehensive clinical trials are needed to confirm their therapeutic value, determine optimal dosages, and assess long-term safety. Incorporating antioxidant-based strategies into personalized treatment plans, particularly for children with ASD, may offer significant benefits in managing behavioral and cognitive symptoms. As scientific understanding deepens, these compounds may become valuable tools in the broader therapeutic landscape of ASD.

## 9 Materials and methods

A structured literature review (PRISMA 2020) was conducted across PubMed, Embase, Cochrane Library, Scopus, and Google Scholar to 31 January 2026. We included preclinical (*in vitro*, *in vivo*) and human clinical studies on ASD that reported either redox biomarkers and/or clinical outcomes. We excluded off-topic articles and studies with insufficient methodological detail (missing model/species, dose, route, duration, controls). Since polyphenols can act as PAINS in biochemical assays (autofluorescence, redox cycling, aggregation, metal chelation), *in-vitro* hits without orthogonal validation were downgraded; *in vivo* and clinical evidence received greater weight. Best-practice compliance for botanical materials. We aligned nomenclature and reporting to ConPhyMP and the Four Pillars, ensuring transparency on botanical drug identity, extract type/characterization (when applicable), and pharmacological context.

Search strategy: “autism spectrum disorder” OR ASD AND oxidative OR redox OR mitochondrial OR neuroinflammation AND “vitamin C”/ascorbate; vitamin E/tocopherol; glutathione/N-acetylcysteine; quercetin; resveratrol; curcumin; crocin/safranal; coenzyme Q10; omega-3; folinic/folate; probiotic/prebiotic.

Eligibility: Preclinical (*in vitro*, *in vivo*) and clinical ASD studies reporting redox biomarkers and/or clinical outcomes; off-topic studies or those lacking model/species, dose/route/duration, or controls were excluded.

Selection and extraction: Two-stage screening (title/abstract; full text). Extraction included model/species, sex, dose/route/duration, tissue, controls, redox biomarkers, clinical outcomes, and safety.

## References

[B1] Ajami M. Pazoki-Toroudi H. Amani H. Nabavi S. F. Braidy N. Vacca R. A. (2017). Therapeutic role of sirtuins in neurodegenerative disease and their modulation by polyphenols. Neurosci. and Biobehav. Rev. 73, 39–47. 10.1016/j.neubiorev.2016.11.022 27914941

[B2] Ak T. Gülçin İ. (2008). Antioxidant and radical scavenging properties of curcumin. Chemico-Biological Interact. 174, 27–37. 10.1016/j.cbi.2008.05.003 18547552

[B3] Al-Askar M. Bhat R. S. Selim M. Al-Ayadhi L. El-Ansary A. (2017). Postnatal treatment using curcumin supplements to amend the damage in VPA-Induced rodent models of autism. BMC Complement. Altern. Med. 17, 259. 10.1186/s12906-017-1763-7 28486989 PMC5424332

[B4] Alfawaz H. Al-Onazi M. Bukhari S. I. Binobead M. Othman N. Algahtani N. (2018). The independent and combined effects of Omega-3 and vitamin B12 in ameliorating propionic acid induced biochemical features in juvenile rats as rodent model of autism. J. Mol. Neurosci. 66, 403–413. 10.1007/s12031-018-1186-z 30284229

[B5] Alinaghi Langari A. Nezhadi A. Kameshki H. Jorjafki S. M. Mirhosseini Y. Khaksari M. (2021). The protective effect of prenatally administered vitamin E on behavioral alterations in an animal model of autism induced by valproic acid. Toxin Rev. 40, 676–680. 10.1080/15569543.2020.1747495

[B6] Alsubaiei S. R. M. Alfawaz H. A. Bhat R. S. El-Ansary A. (2023). Nutritional intervention as a complementary neuroprotective approach against propionic acid-induced neurotoxicity and associated biochemical autistic features in rat pups. Metabolites 13, 738. 10.3390/metabo13060738 37367896 PMC10302727

[B7] Arndt T. L. Stodgell C. J. Rodier P. M. (2005). The teratology of autism. Intl J Devlp Neurosci. 23, 189–199. 10.1016/j.ijdevneu.2004.11.001 15749245

[B8] Ashwood P. Van De Water J. (2004). Is autism an autoimmune disease? Autoimmun. Rev. 3, 557–562. 10.1016/j.autrev.2004.07.036 15546805

[B9] Ashwood P. Krakowiak P. Hertz-Picciotto I. Hansen R. Pessah I. Van De Water J. (2011). Elevated plasma cytokines in autism spectrum disorders provide evidence of immune dysfunction and are associated with impaired behavioral outcome. Brain, Behav. Immun. 25, 40–45. 10.1016/j.bbi.2010.08.003 20705131 PMC2991432

[B10] Badawi N. Dixon G. S. Felix J. F. Keogh J. M. Petterson B. Stanley F. J. (2006). Autism following a history of newborn encephalopathy: more than a coincidence? Develop. Med. Child. Neuro 48, 85–89. 10.1017/S001216220600020X 16417661

[B11] Baell J. B. (2016). Feeling nature’s PAINS: natural products, natural product drugs, and pan assay interference compounds (PAINS). J. Nat. Prod. 79, 616–628. 10.1021/acs.jnatprod.5b00947 26900761

[B12] Bambini-Junior V. Zanatta G. Della Flora Nunes G. Mueller De Melo G. Michels M. Fontes-Dutra M. (2014). Resveratrol prevents social deficits in animal model of autism induced by valproic acid. Neurosci. Lett. 583, 176–181. 10.1016/j.neulet.2014.09.039 25263788

[B13] Bej E. Cesare P. d’Angelo M. Volpe A. R. Castelli V. (2024a). Neuronal cell rearrangement during aging: Antioxidant compounds as a potential therapeutic approach. Cells 13, 1945. 10.3390/cells13231945 39682694 PMC11639796

[B14] Bej E. Cesare P. Volpe A. R. d’Angelo M. Castelli V. (2024b). Oxidative stress and neurodegeneration: insights and therapeutic strategies for parkinson’s disease. Neurol. Int. 16, 502–517. 10.3390/neurolint16030037 38804477 PMC11130796

[B15] Bej E. Volpe A. R. Cesare P. Cimini A. d’Angelo M. Castelli V. (2024c). Therapeutic potential of saffron in brain disorders: from bench to bedside. Phytother. Res. 38, 2482–2495. 10.1002/ptr.8169 38446350

[B16] Bhandari R. Kuhad A. (2015). Neuropsychopharmacotherapeutic efficacy of curcumin in experimental paradigm of autism spectrum disorders. Life Sci. 141, 156–169. 10.1016/j.lfs.2015.09.012 26407474

[B17] Bhandari R. Kuhad A. (2017). Resveratrol suppresses neuroinflammation in the experimental paradigm of autism spectrum disorders. Neurochem. Int. 103, 8–23. 10.1016/j.neuint.2016.12.012 28025035

[B18] Billeci L. Callara A. L. Guiducci L. Prosperi M. Morales M. A. Calderoni S. (2023). A randomized controlled trial into the effects of probiotics on electroencephalography in preschoolers with autism. Autism 27, 117–132. 10.1177/13623613221082710 35362336 PMC9806478

[B19] Bin-Khattaf R. M. Al-Dbass A. M. Alonazi M. Bhat R. S. Al-Daihan S. El-Ansary A. K. (2024). In a rodent model of autism, probiotics decrease gut leakiness in relation to gene expression of GABA receptors: emphasize how crucial the gut-brain axis. Transl. Neurosci. 15, 20220354. 10.1515/tnsci-2022-0354 39380963 PMC11459612

[B20] Bjørklund G. Waly M. I. Al-Farsi Y. Saad K. Dadar M. Rahman M. M. (2019). The role of vitamins in autism spectrum disorder: what do we know? J. Mol. Neurosci. 67, 373–387. 10.1007/s12031-018-1237-5 30607900

[B21] Bjørklund G. Doşa M. D. Maes M. Dadar M. Frye R. E. Peana M. (2021). The impact of glutathione metabolism in autism spectrum disorder. Pharmacol. Res. 166, 105437. 10.1016/j.phrs.2021.105437 33493659

[B22] Bloomfield P. S. Selvaraj S. Veronese M. Rizzo G. Bertoldo A. Owen D. R. (2016). Microglial activity in people at ultra high risk of psychosis and in schizophrenia: an [^11^ C]PBR28 PET brain imaging study. AJP 173, 44–52. 10.1176/appi.ajp.2015.14101358 26472628 PMC4821370

[B23] Bolz S. N. Adasme M. F. Schroeder M. (2021). Toward an understanding of pan-assay interference compounds and promiscuity: a structural perspective on binding modes. J. Chem. Inf. Model. 61, 2248–2262. 10.1021/acs.jcim.0c01227 33899463

[B24] Boone K. M. Klebanoff M. A. Rogers L. K. Rausch J. Coury D. L. Keim S. A. (2022). Effects of Omega-3-6-9 fatty acid supplementation on behavior and sleep in preterm toddlers with autism symptomatology: secondary analysis of a randomized clinical trial. Early Hum. Dev. 169, 105588. 10.1016/j.earlhumdev.2022.105588 35644107 PMC9516351

[B25] Braunschweig D. Van De Water J. (2012). Maternal autoantibodies in autism. Arch. Neurol. 69, 693–699. 10.1001/archneurol.2011.2506 22689191 PMC4776649

[B26] Brimberg L. Sadiq A. Gregersen P. K. Diamond B. (2013). Brain-reactive IgG correlates with autoimmunity in mothers of a child with an autism spectrum disorder. Mol. Psychiatry 18, 1171–1177. 10.1038/mp.2013.101 23958959

[B27] Castejon A. M. Spaw J. A. Rozenfeld I. Sheinberg N. Kabot S. Shaw A. (2021). Improving antioxidant capacity in children with autism: a randomized, double-blind controlled study with cysteine-rich whey protein. Front. Psychiatry 12, 669089. 10.3389/fpsyt.2021.669089 34658941 PMC8514994

[B28] Castelli V. Grassi D. Bocale R. d’Angelo M. Antonosante A. Cimini A. (2018). Diet and brain health: which role for polyphenols? CPD 24, 227–238. 10.2174/1381612824666171213100449 29237377

[B29] Chauhan A. Chauhan V. Brown W. T. Cohen I. (2004). Oxidative stress in autism: increased lipid peroxidation and reduced serum levels of ceruloplasmin and transferrin - the antioxidant proteins. Life Sci. 75, 2539–2549. 10.1016/j.lfs.2004.04.038 15363659

[B30] Chauhan A. Gu F. Essa M. M. Wegiel J. Kaur K. Brown W. T. (2011). Brain region‐specific deficit in mitochondrial electron transport chain complexes in children with autism. J. Neurochem. 117, 209–220. 10.1111/j.1471-4159.2011.07189.x 21250997 PMC4839269

[B31] Chauhan A. Audhya T. Chauhan V. (2012). Brain region-specific glutathione redox imbalance in autism. Neurochem. Res. 37, 1681–1689. 10.1007/s11064-012-0775-4 22528835

[B32] Chełchowska M. Gajewska J. Szczepanik E. Mazur J. Cychol A. Kuźniar-Pałka A. (2025). Oxidative stress indicated by nuclear transcription factor Nrf2 and glutathione status in the blood of young children with autism spectrum disorder: pilot study. Antioxidants (Basel) 14, 320. 10.3390/antiox14030320 40227289 PMC11939242

[B166] Chen H. Yang T. Chen J. Chen L. Dai Y. Zhang J. (2021). Sleep problems in children with autism spectrum disorder: a multicenter survey. BMC psychiatry 21 (1), 406. 10.1186/s12888-021-03405-w 34399715 PMC8365936

[B33] Courchesne E. Karns C. M. Davis H. R. Ziccardi R. Carper R. A. Tigue Z. D. (2001). Unusual brain growth patterns in early life in patients with autistic disorder: an MRI study. Neurology 57, 245–254. 10.1212/WNL.57.2.245 11468308

[B34] Croen L. A. Najjar D. V. Fireman B. Grether J. K. (2007). Maternal and paternal age and risk of autism spectrum disorders. Arch. Pediatr. Adolesc. Med. 161, 334–340. 10.1001/archpedi.161.4.334 17404129

[B35] Croen L. A. Braunschweig D. Haapanen L. Yoshida C. K. Fireman B. Grether J. K. (2008). Maternal mid-pregnancy autoantibodies to fetal brain protein: the early markers for autism study. Biol. Psychiatry 64, 583–588. 10.1016/j.biopsych.2008.05.006 18571628 PMC2574992

[B36] Cucinotta F. Ricciardello A. Turriziani L. Mancini A. Keller R. Sacco R. (2022). Efficacy and safety of Q10 ubiquinol with vitamins B and E in neurodevelopmental disorders: a retrospective chart review. Front. Psychiatry 13, 829516. 10.3389/fpsyt.2022.829516 35308885 PMC8927903

[B37] Davinelli S. Medoro A. Siracusano M. Savino R. Saso L. Scapagnini G. (2025). Oxidative stress response and NRF2 signaling pathway in autism spectrum disorder. Redox Biol. 83, 103661. 10.1016/j.redox.2025.103661 40324316 PMC12099462

[B38] De Magistris L. Picardi A. Siniscalco D. Riccio M. P. Sapone A. Cariello R. (2013). Antibodies against food antigens in patients with autistic spectrum disorders. BioMed Res. Int. 2013, 1–11. 10.1155/2013/729349 23984403 PMC3747333

[B39] De Mattos B. D. S. Soares M. S. P. Spohr L. Pedra N. S. Teixeira F. C. De Souza A. A. (2020). Quercetin prevents alterations of behavioral parameters, delta‐aminolevulinic dehydratase activity, and oxidative damage in brain of rats in a prenatal model of autism. Intl J Devlp Neurosci. 80, 287–302. 10.1002/jdn.10025 32181519

[B40] Dellapiazza F. Michelon C. Rattaz C. Picot M.-C. Baghdadli A. (2022). Sex-related differences in clinical characteristics of children with ASD without ID: results from the ELENA cohort. Front. Psychiatry 13, 998195. 10.3389/fpsyt.2022.998195 36518364 PMC9742240

[B41] Depino A. M. (2013). Peripheral and central inflammation in autism spectrum disorders. Mol. Cell. Neurosci. 53, 69–76. 10.1016/j.mcn.2012.10.003 23069728

[B42] Doaei S. Bourbour F. Teymoori Z. Jafari F. Kalantari N. Abbas Torki S. (2021). The effect of omega-3 fatty acids supplementation on social and behavioral disorders of children with autism: a randomized clinical trial. Pedm 27, 12–18. 10.5114/pedm.2020.101806 33599431 PMC10227477

[B167] Doherty M. Neilson S. O’Sullivan J. Carravallah L. Johnson M. Cullen W. (2022). Barriers to healthcare and self-reported adverse outcomes for autistic adults: a cross-sectional study. BMJ open 12 (2), e056904. 10.1136/bmjopen-2021-056904 35193921 PMC8883251

[B43] Dong S. Zeng Q. Mitchell E. S. Xiu J. Duan Y. Li C. (2012). Curcumin enhances neurogenesis and cognition in aged rats: implications for transcriptional interactions related to growth and synaptic plasticity. PLoS ONE 7, e31211. 10.1371/journal.pone.0031211 22359574 PMC3281036

[B44] El-Ansary A. Bjørklund G. Khemakhem A. M. Al-Ayadhi L. Chirumbolo S. Ben Bacha A. (2018). Metabolism-associated markers and childhood autism rating scales (CARS) as a measure of autism severity. J. Mol. Neurosci. 65, 265–276. 10.1007/s12031-018-1091-5 29931502

[B45] El-Demerdash F. M. Yousef M. I. Radwan F. M. E. (2009). Ameliorating effect of curcumin on sodium arsenite-induced oxidative damage and lipid peroxidation in different rat organs. Food Chem. Toxicol. 47, 249–254. 10.1016/j.fct.2008.11.013 19049818

[B46] Ellul P. Rosenzwajg M. Peyre H. Fourcade G. Mariotti-Ferrandiz E. Trebossen V. (2021). Regulatory T lymphocytes/Th17 lymphocytes imbalance in autism spectrum disorders: evidence from a meta-analysis. Mol. Autism 12, 68. 10.1186/s13229-021-00472-4 34641964 PMC8507168

[B47] Emanuele E. Orsi P. Boso M. Broglia D. Brondino N. Barale F. (2010). Low-grade endotoxemia in patients with severe autism. Neurosci. Lett. 471, 162–165. 10.1016/j.neulet.2010.01.033 20097267

[B48] Enstrom A. M. Onore C. E. Van De Water J. A. Ashwood P. (2010). Differential monocyte responses to TLR ligands in children with autism spectrum disorders. Brain, Behav. Immun. 24, 64–71. 10.1016/j.bbi.2009.08.001 19666104 PMC3014091

[B49] Faja S. Dawson G. (2017). “Autism spectrum disorders,” in Child and adolescent psychopathology. Editors Beauchaine T. P. Hinshaw S. P. Third Edition (Wiley), 745–782. 10.1002/9781394258932.ch22

[B50] Fernandes A. Miller-Fleming L. Pais T. F. (2014). Microglia and inflammation: conspiracy, controversy or control? Cell. Mol. Life Sci. 71, 3969–3985. 10.1007/s00018-014-1670-8 25008043 PMC11113719

[B51] Figueroa-Méndez R. Rivas-Arancibia S. (2015). Vitamin C in health and disease: its role in the metabolism of cells and redox state in the brain. Front. Physiol. 6, 397. 10.3389/fphys.2015.00397 26779027 PMC4688356

[B52] Filipek P. A. Juranek J. Nguyen M. T. Cummings C. Gargus J. J. (2004). Relative carnitine deficiency in autism. J. Autism Dev. Disord. 34, 615–623. 10.1007/s10803-004-5283-1 15679182

[B53] Fiorentino M. Sapone A. Senger S. Camhi S. S. Kadzielski S. M. Buie T. M. (2016). Blood–brain barrier and intestinal epithelial barrier alterations in autism spectrum disorders. Mol. Autism 7, 49. 10.1186/s13229-016-0110-z 27957319 PMC5129651

[B54] Friedman S. D. Shaw D. W. Artru A. A. Richards T. L. Gardner J. Dawson G. (2003). Regional brain chemical alterations in young children with autism spectrum disorder. Neurology 60, 100–107. 10.1212/WNL.60.1.100 12525726

[B55] Frustaci A. Neri M. Cesario A. Adams J. B. Domenici E. Dalla Bernardina B. (2012). Oxidative stress-related biomarkers in autism: systematic review and meta-analyses. Free Radic. Biol. Med. 52, 2128–2141. 10.1016/j.freeradbiomed.2012.03.011 22542447

[B56] Frye R. E. (2020). Mitochondrial dysfunction in autism spectrum disorder: unique abnormalities and targeted treatments. Seminars Pediatr. Neurology 35, 100829. 10.1016/j.spen.2020.100829 32892956

[B57] Frye R. E. Slattery J. Delhey L. Furgerson B. Strickland T. Tippett M. (2018). Folinic acid improves verbal communication in children with autism and language impairment: a randomized double-blind placebo-controlled trial. Mol. Psychiatry 23, 247–256. 10.1038/mp.2016.168 27752075 PMC5794882

[B58] Frye R. E. Rincon N. McCarty P. J. Brister D. Scheck A. C. Rossignol D. A. (2024). Biomarkers of mitochondrial dysfunction in autism spectrum disorder: a systematic review and meta-analysis. Neurobiol. Dis. 197, 106520. 10.1016/j.nbd.2024.106520 38703861

[B59] Garland E. F. Hartnell I. J. Boche D. (2022). Microglia and astrocyte function and communication: what do we know in humans? Front. Neurosci. 16, 824888. 10.3389/fnins.2022.824888 35250459 PMC8888691

[B60] Gevezova M. Sbirkov Y. Sarafian V. Plaimas K. Suratanee A. Maes M. (2023). Autistic spectrum disorder (ASD) - gene, molecular and pathway signatures linking systemic inflammation, mitochondrial dysfunction, transsynaptic signalling, and neurodevelopment. Brain Behav. Immun. Health 30, 100646. 10.1016/j.bbih.2023.100646 37334258 PMC10275703

[B61] Ghanizadeh A. Akhondzadeh S. Hormozi M. Makarem A. Abotorabi-Zarchi M. Firoozabadi A. (2012). Glutathione-related factors and oxidative stress in autism. A Rev. CMC 19, 4000–4005. 10.2174/092986712802002572 22708999

[B62] Grimaldi R. Gibson G. R. Vulevic J. Giallourou N. Castro-Mejía J. L. Hansen L. H. (2018). A prebiotic intervention study in children with autism spectrum disorders (ASDs). Microbiome 6, 133. 10.1186/s40168-018-0523-3 30071894 PMC6091020

[B63] Gu F. Chauhan V. Kaur K. Brown W. T. LaFauci G. Wegiel J. (2013). Alterations in mitochondrial DNA copy number and the activities of electron transport chain complexes and pyruvate dehydrogenase in the frontal cortex from subjects with autism. Transl. Psychiatry 3, e299. 10.1038/tp.2013.68 24002085 PMC3784762

[B64] Haas R. H. Parikh S. Falk M. J. Saneto R. P. Wolf N. I. Darin N. (2007). Mitochondrial disease: a practical approach for primary care physicians. Pediatrics 120, 1326–1333. 10.1542/peds.2007-0391 18055683

[B65] Han V. X. Patel S. Jones H. F. Dale R. C. (2021). Maternal immune activation and neuroinflammation in human neurodevelopmental disorders. Nat. Rev. Neurol. 17, 564–579. 10.1038/s41582-021-00530-8 34341569

[B66] Hazlett H. C. Poe M. D. Gerig G. Styner M. Chappell C. Smith R. G. (2011). Early brain overgrowth in autism associated with an increase in cortical surface area before age 2 years. Arch. Gen. Psychiatry 68, 467–476. 10.1001/archgenpsychiatry.2011.39 21536976 PMC3315057

[B67] Hendouei F. Sanjari Moghaddam H. Mohammadi M. R. Taslimi N. Rezaei F. Akhondzadeh S. (2020). Resveratrol as adjunctive therapy in treatment of irritability in children with autism: a double‐blind and placebo‐controlled randomized trial. J. Clin. Pharm. Ther. 45, 324–334. 10.1111/jcpt.13076 31714621

[B68] Hill Z. McCarty P. J. Boles R. G. Frye R. E. (2025). A mitochondrial supplement improves function and mitochondrial activity in autism: a double-blind placebo-controlled cross-over trial. Int. J. Mol. Sci. 26, 2479. 10.3390/ijms26062479 40141123 PMC11941969

[B69] Hirayama A. Wakusawa K. Fujioka T. Iwata K. Usui N. Kurita D. (2020). Simultaneous evaluation of antioxidative serum profiles facilitates the diagnostic screening of autism spectrum disorder in under-6-year-old children. Sci. Rep. 10, 20602. 10.1038/s41598-020-77328-z 33244118 PMC7691362

[B70] Hosseini S. Ghadimi M. Reyhani N. Khazaei S. Rahmatkhah-Yazdi M. Soleimani-Farsani R. (2025). BDNF and GSK-3beta expression changes underlie the beneficial effects of crocin on behavioral alterations in a rat model of autism induced by prenatal valproic acid administration. Naunyn Schmiedeb. Arch. Pharmacol. 398, 7571–7582. 10.1007/s00210-024-03777-2 39777538

[B71] Hu T. Dong Y. He C. Zhao M. He Q. (2020). The gut microbiota and oxidative stress in autism spectrum disorders (ASD). Oxidative Med. Cell. Longev. 2020, 1–13. 10.1155/2020/8396708 33062148 PMC7547345

[B72] Inga Jácome M. Morales Chacòn L. Vera Cuesta H. Maragoto Rizo C. Whilby Santiesteban M. Ramos Hernandez L. (2016). Peripheral inflammatory markers contributing to comorbidities in autism. Behav. Sci. 6, 29. 10.3390/bs6040029 27983615 PMC5197942

[B73] Israelyan N. Margolis K. G. (2018). Serotonin as a link between the gut-brain-microbiome axis in autism spectrum disorders. Pharmacol. Res. 132, 1–6. 10.1016/j.phrs.2018.03.020 29614380 PMC6368356

[B74] Izzo C. Annunziata M. Melara G. Sciorio R. Dallio M. Masarone M. (2021). The role of resveratrol in liver disease: a comprehensive review from *in vitro* to clinical trials. Nutrients 13, 933. 10.3390/nu13030933 33805795 PMC7999728

[B75] James S. J. Cutler P. Melnyk S. Jernigan S. Janak L. Gaylor D. W. (2004). Metabolic biomarkers of increased oxidative stress and impaired methylation capacity in children with autism. Am. J. Clin. Nutr. 80, 1611–1617. 10.1093/ajcn/80.6.1611 15585776

[B76] James S. J. Rose S. Melnyk S. Jernigan S. Blossom S. Pavliv O. (2009). Cellular and mitochondrial glutathione redox imbalance in lymphoblastoid cells derived from children with autism. FASEB J. 23, 2374–2383. 10.1096/fj.08-128926 19307255 PMC2717775

[B77] Jardim F. R. De Rossi F. T. Nascimento M. X. Da Silva Barros R. G. Borges P. A. Prescilio I. C. (2018). Resveratrol and brain mitochondria: a review. Mol. Neurobiol. 55, 2085–2101. 10.1007/s12035-017-0448-z 28283884

[B78] Jayaprakash P. Isaev D. Shabbir W. Lorke D. E. Sadek B. Oz M. (2021). Curcumin potentiates α7 nicotinic acetylcholine receptors and alleviates autistic-like social deficits and brain oxidative stress status in mice. Int. J. Mol. Sci. 22, 7251. 10.3390/ijms22147251 34298871 PMC8303708

[B79] Johnson C. P. Myers S. M. the Council on Children With Disabilities (2007). Identification and evaluation of children with autism spectrum disorders. Pediatrics 120, 1183–1215. 10.1542/peds.2007-2361 17967920

[B80] Kato Y. Yokokura M. Iwabuchi T. Murayama C. Harada T. Goto T. (2023). Lower availability of mitochondrial complex I in anterior cingulate cortex in autism: a positron emission tomography study. AJP 180, 277–284. 10.1176/appi.ajp.22010014 36069020

[B81] Kern J. K. Geier D. A. Adams J. B. Garver C. R. Audhya T. Geier M. R. (2011). A clinical trial of glutathione supplementation in autism spectrum disorders. Med. Sci. Monit. 17, CR677–CR682. 10.12659/MSM.882125 22129897 PMC3628138

[B82] Kong Q. Wang B. Tian P. Li X. Zhao J. Zhang H. (2021). Daily intake of *lactobacillus* alleviates autistic-like behaviors by ameliorating the 5-hydroxytryptamine metabolic disorder in VPA-Treated rats during weaning and sexual maturation. Food Funct. 12, 2591–2604. 10.1039/D0FO02375B 33629689

[B83] Kong X.-J. Liu J. Liu K. Koh M. Sherman H. Liu S. (2021). Probiotic and oxytocin combination therapy in patients with autism spectrum disorder: a randomized, double-blinded, placebo-controlled pilot trial. Nutrients 13, 1552. 10.3390/nu13051552 34062986 PMC8147925

[B84] Kothari P. Tate A. Adewumi A. Kinlin L. M. Ritwik P. (2020). The risk for scurvy in children with neurodevelopmental disorders. Special Care Dent. 40, 251–259. 10.1111/scd.12459 32330999

[B85] Krey J. F. Dolmetsch R. E. (2007). Molecular mechanisms of autism: a possible role for Ca2+ signaling. Curr. Opin. Neurobiol. 17, 112–119. 10.1016/j.conb.2007.01.010 17275285

[B86] Krishnamurthy H. K. Rajavelu I. Pereira M. Jayaraman V. Krishna K. Wang T. (2024). Inside the genome: understanding genetic influences on oxidative stress. Front. Genet. 15, 1397352. 10.3389/fgene.2024.1397352 38983269 PMC11231378

[B87] Kulkarni A. Wilson D. M. (2008). The involvement of DNA-damage and -Repair defects in neurological dysfunction. Am. J. Hum. Genet. 82, 539–566. 10.1016/j.ajhg.2008.01.009 18319069 PMC2427185

[B88] Larbi A. Kempf J. Pawelec G. (2007). Oxidative stress modulation and T cell activation. Exp. Gerontol. 42, 852–858. 10.1016/j.exger.2007.05.004 17604927

[B89] Li X. Chauhan A. Sheikh A. M. Patil S. Chauhan V. Li X.-M. (2009). Elevated immune response in the brain of autistic patients. J. Neuroimmunol. 207, 111–116. 10.1016/j.jneuroim.2008.12.002 19157572 PMC2770268

[B90] Li Y. Yao J. Han C. Yang J. Chaudhry M. Wang S. (2016). Quercetin, inflammation and immunity. Nutrients 8, 167. 10.3390/nu8030167 26999194 PMC4808895

[B91] Lin X. Zhou Y. Li S. Zhou H. Ma B. Zhang Z. (2022). Markers related to oxidative stress in peripheral blood in children with autism spectrum disorder. Res. Autism Spectr. Disord. 99, 102067. 10.1016/j.rasd.2022.102067

[B92] Lord C. Elsabbagh M. Baird G. Veenstra-Vanderweele J. (2018). Autism spectrum disorder. Lancet 392, 508–520. 10.1016/S0140-6736(18)31129-2 30078460 PMC7398158

[B93] Lubbers K. Hiralal K. R. Dieleman G. C. Hagenaar D. A. Dierckx B. Legerstee J. S. (2024). Autism spectrum disorder symptom profiles in fragile X syndrome, angelman syndrome, Tuberous sclerosis complex and neurofibromatosis type 1. J. Autism Dev. Disord. 56, 793–807. 10.1007/s10803-024-06557-2 39395123 PMC12864356

[B94] Majerczyk D. Ayad E. G. Brewton K. L. Saing P. Hart P. C. (2022). Systemic maternal inflammation promotes ASD *via* IL-6 and IFN-γ. Biosci. Rep. 42, BSR20220713. 10.1042/BSR20220713 36300375 PMC9670245

[B95] Malaguarnera M. Khan H. Cauli O. (2020). Resveratrol in autism spectrum disorders: behavioral and molecular effects. Antioxidants 9, 188. 10.3390/antiox9030188 32106489 PMC7139867

[B96] Manivasagam T. Arunadevi S. Essa M. M. SaravanaBabu C. Borah A. Thenmozhi A. J. (2020). “Role of oxidative stress and antioxidants in autism,” in Personalized food intervention and therapy for autism spectrum disorder management, advances in neurobiology. Editors Essa M. M. Qoronfleh M. W. (Cham: Springer International Publishing), 193–206. 10.1007/978-3-030-30402-7_7 32006361

[B97] Margedari P. Goudarzi I. Sepehri H. (2024). The protective role of prenatal administration of ascorbic acid on autistic-like behavior in a rat model of autism. IBRO Neurosci. Rep. 16, 78–85. 10.1016/j.ibneur.2023.11.002 38274439 PMC10809097

[B98] Marí-Bauset S. Llopis-González A. Zazpe-García I. Marí-Sanchis A. Morales-Suárez-Varela M. (2015). Nutritional status of children with autism spectrum disorders (ASDs): a case–control study. J. Autism Dev. Disord. 45, 203–212. 10.1007/s10803-014-2205-8 25194628

[B99] McCaffery P. Deutsch C. K. (2005). Macrocephaly and the control of brain growth in autistic disorders. Prog. Neurobiol. 77, 38–56. 10.1016/j.pneurobio.2005.10.005 16280193

[B100] Meguid N. A. Atta H. M. Gouda A. S. Khalil R. O. (2008). Role of polyunsaturated fatty acids in the management of Egyptian children with autism. Clin. Biochem. 41, 1044–1048. 10.1016/j.clinbiochem.2008.05.013 18582451

[B101] Moaaz M. Youssry S. Elfatatry A. El Rahman M. A. (2019). Th17/Treg cells imbalance and their related cytokines (IL-17, IL-10 and TGF-β) in children with autism spectrum disorder. J. Neuroimmunol. 337, 577071. 10.1016/j.jneuroim.2019.577071 31671361

[B102] Moghaddam A. H. Eslami A. Jelodar S. K. Ranjbar M. Hasantabar V. (2023). Preventive effect of quercetin-Loaded nanophytosome against autistic-like damage in maternal separation model: the possible role of Caspase-3, Bax/Bcl-2 and Nrf2. Behav. Brain Res. 441, 114300. 10.1016/j.bbr.2023.114300 36642103

[B103] Mousavinejad E. Ghaffari M. A. Riahi F. Hajmohammadi M. Tiznobeyk Z. Mousavinejad M. (2018). Coenzyme Q10 supplementation reduces oxidative stress and decreases antioxidant enzyme activity in children with autism spectrum disorders. Psychiatry Res. 265, 62–69. 10.1016/j.psychres.2018.03.061 29684771

[B104] Moussa C. Hebron M. Huang X. Ahn J. Rissman R. A. Aisen P. S. (2017). Resveratrol regulates neuro-inflammation and induces adaptive immunity in Alzheimer’s disease. J. Neuroinflammation 14, 1. 10.1186/s12974-016-0779-0 28086917 PMC5234138

[B105] Napolitano A. Schiavi S. La Rosa P. Rossi-Espagnet M. C. Petrillo S. Bottino F. (2022). Sex differences in autism spectrum disorder: diagnostic, neurobiological, and behavioral features. Front. Psychiatry 13, 889636. 10.3389/fpsyt.2022.889636 35633791 PMC9136002

[B106] Nathan C. Cunningham-Bussel A. (2013). Beyond oxidative stress: an immunologist’s guide to reactive oxygen species. Nat. Rev. Immunol. 13, 349–361. 10.1038/nri3423 23618831 PMC4250048

[B107] Navarro F. Pearson D. A. Fatheree N. Mansour R. Hashmi S. S. Rhoads J. M. (2015). Are ‘leaky gut’ and behavior associated with gluten and dairy containing diet in children with autism spectrum disorders? Nutr. Neurosci. 18, 177–185. 10.1179/1476830514Y.0000000110 24564346

[B108] Navarro-Cruz A. R. Ramírez Y Ayala R. Ochoa-Velasco C. Brambila E. Avila-Sosa R. Pérez-Fernández S. (2017). Effect of chronic administration of resveratrol on cognitive performance during aging process in rats. Oxidative Med. Cell. Longev. 2017, 8510761. 10.1155/2017/8510761 29163756 PMC5661096

[B109] Neggers Y. (2014). The relationship between folic acid and risk of autism spectrum disorders. Healthcare 2, 429–444. 10.3390/healthcare2040429 27429286 PMC4934568

[B110] Nelson K. B. Grether J. K. Croen L. A. Dambrosia J. M. Dickens B. F. Jelliffe L. L. (2001). Neuropeptides and neurotrophins in neonatal blood of children with autism or mental retardation. Ann. Neurology 49, 597–606. 10.1002/ana.1024 11357950

[B111] Nelson K. M. Dahlin J. L. Bisson J. Graham J. Pauli G. F. Walters M. A. (2017). The essential medicinal chemistry of curcumin: miniperspective. J. Med. Chem. 60, 1620–1637. 10.1021/acs.jmedchem.6b00975 28074653 PMC5346970

[B112] Oliveira G. Diogo L. Grazina M. Garcia P. Ataíde A. Marques C. (2005). Mitochondrial dysfunction in autism spectrum disorders: a population-based study. Dev. Med. Child. Neurol. 47, 185–189. 10.1017/S0012162205000332 15739723

[B113] Palmieri L. Persico A. M. (2010). Mitochondrial dysfunction in autism spectrum disorders: cause or effect? Biochimica Biophysica Acta (BBA) - Bioenergetics 1797, 1130–1137. 10.1016/j.bbabio.2010.04.018 20441769

[B114] Pangrazzi L. Balasco L. Bozzi Y. (2020a). Oxidative stress and immune system dysfunction in autism spectrum disorders. IJMS 21, 3293. 10.3390/ijms21093293 32384730 PMC7247582

[B115] Pangrazzi L. Balasco L. Bozzi Y. (2020b). Natural antioxidants: a novel therapeutic approach to autism spectrum disorders? Antioxidants 9, 1186. 10.3390/antiox9121186 33256243 PMC7761361

[B116] Pastural É. Ritchie S. Lu Y. Jin W. Kavianpour A. Khine Su-Myat K. (2009). Novel plasma phospholipid biomarkers of autism: mitochondrial dysfunction as a putative causative mechanism. Prostagl. Leukot. Essent. Fat. Acids 81, 253–264. 10.1016/j.plefa.2009.06.003 19608392

[B117] Patterson P. H. (2009). Immune involvement in schizophrenia and autism: etiology, pathology and animal models. Behav. Brain Res. 204, 313–321. 10.1016/j.bbr.2008.12.016 19136031

[B118] Pinto Payares D. V. Spooner L. Vosters J. Dominguez S. Patrick L. Harris A. (2024). A systematic review on the role of mitochondrial dysfunction/disorders in neurodevelopmental disorders and psychiatric/behavioral disorders. Front. Psychiatry 15, 1389093. 10.3389/fpsyt.2024.1389093 39006821 PMC11239503

[B119] Pisoschi A. M. Pop A. (2015). The role of antioxidants in the chemistry of oxidative stress: a review. Eur. J. Med. Chem. 97, 55–74. 10.1016/j.ejmech.2015.04.040 25942353

[B120] Poling J. S. Frye R. E. Shoffner J. Zimmerman A. W. (2006). Developmental regression and mitochondrial dysfunction in a child with autism. J. Child. Neurol. 21, 170–172. 10.1177/08830738060210021401 16566887 PMC2536523

[B121] Politi P. Cena H. Comelli M. Marrone G. Allegri C. Emanuele E. (2008). Behavioral effects of Omega-3 fatty acid supplementation in young adults with severe autism: an open label study. Archives Med. Res. 39, 682–685. 10.1016/j.arcmed.2008.06.005 18760197

[B122] Prata J. Machado A. S. Von Doellinger O. Almeida M. I. Barbosa M. A. Coelho R. (2019). “The contribution of inflammation to autism spectrum disorders: recent clinical evidence,” in Psychiatric disorders, methods in molecular biology. Editor Kobeissy F. H. (New York, New York, NY: Springer), 493–510. 10.1007/978-1-4939-9554-7_29 31273718

[B123] Qiu S. Li L. Weeber E. J. May J. M. (2007). Ascorbate transport by primary cultured neurons and its role in neuronal function and protection against excitotoxicity. J Neurosci. Res. 85, 1046–1056. 10.1002/jnr.21204 17304569

[B124] Ramaekers V. Blau N. Sequeira J. Nassogne M.-C. Quadros E. (2007). Folate receptor autoimmunity and cerebral folate deficiency in low-functioning autism with neurological deficits. Neuropediatrics 38, 276–281. 10.1055/s-2008-1065354 18461502

[B125] Ramaekers V. T. Quadros E. V. Sequeira J. M. (2013). Role of folate receptor autoantibodies in infantile autism. Mol. Psychiatry 18, 270–271. 10.1038/mp.2012.22 22488256

[B126] Raymond L. J. Deth R. C. Ralston N. V. C. (2014). Potential role of selenoenzymes and antioxidant metabolism in relation to autism etiology and pathology. Autism Res. Treat. 2014, 1–15. 10.1155/2014/164938 24734177 PMC3966422

[B127] Renard E. Leheup B. Guéant-Rodriguez R.-M. Oussalah A. Quadros E. V. Guéant J.-L. (2020). Folinic acid improves the score of autism in the EFFET placebo-controlled randomized trial. Biochimie 173, 57–61. 10.1016/j.biochi.2020.04.019 32387472

[B128] Rose S. Melnyk S. Savenka A. Hubanks A. Cleves S. J. M. James S. J. (2008). The frequency of polymorphisms affecting lead and mercury toxicity among children with autism. Am. J. Biochem. Biotechnol. 4, 85–94. 10.3844/ajbbsp.2008.85.94

[B129] Rose S. Niyazov D. M. Rossignol D. A. Goldenthal M. Kahler S. G. Frye R. E. (2018). Clinical and molecular characteristics of mitochondrial dysfunction in autism spectrum disorder. Mol. Diagn Ther. 22, 571–593. 10.1007/s40291-018-0352-x 30039193 PMC6132446

[B130] Rossignol D. A. Frye R. E. (2012). Mitochondrial dysfunction in autism spectrum disorders: a systematic review and meta-analysis. Mol. Psychiatry 17, 290–314. 10.1038/mp.2010.136 21263444 PMC3285768

[B131] Rossignol D. A. Frye R. E. (2021). Cerebral folate deficiency, folate receptor alpha autoantibodies and leucovorin (folinic acid) treatment in autism spectrum disorders: a systematic review and meta-analysis. JPM 11, 1141. 10.3390/jpm11111141 34834493 PMC8622150

[B132] Rushworth G. F. Megson I. L. (2014). Existing and potential therapeutic uses for N-acetylcysteine: the need for conversion to intracellular glutathione for antioxidant benefits. Pharmacol. and Ther. 141, 150–159. 10.1016/j.pharmthera.2013.09.006 24080471

[B133] Russo F. B. Freitas B. C. Pignatari G. C. Fernandes I. R. Sebat J. Muotri A. R. (2018). Modeling the interplay between neurons and astrocytes in autism using human induced pluripotent stem cells. Biol. Psychiatry 83, 569–578. 10.1016/j.biopsych.2017.09.021 29129319

[B134] Sachdeva P. Mehdi I. Kaith R. Ahmad F. Anwar M. S. (2022). Potential natural products for the management of autism spectrum disorder. Ibrain 8, 365–376. 10.1002/ibra.12050 37786737 PMC10528773

[B135] Sakamornchai W. Dumrongwongsiri O. Siwarom S. (2022). Case report: vitamin C combined with multiple micronutrient deficiencies is associated with pulmonary arterial hypertension in children with autistic spectrum disorder. Front. Nutr. 9, 928026. 10.3389/fnut.2022.928026 36337659 PMC9631921

[B136] Salcedo-Arellano M. J. Cabal-Herrera A. M. Punatar R. H. Clark C. J. Romney C. A. Hagerman R. J. (2021). Overlapping molecular pathways leading to autism spectrum disorders, fragile X syndrome, and targeted treatments. Neurotherapeutics 18, 265–283. 10.1007/s13311-020-00968-6 33215285 PMC8116395

[B168] Santocchi E. Guiducci L. Prosperi M. Calderoni S. Gaggini M. Apicella F. (2020). Effects of probiotic supplementation on gastrointestinal, sensory and core symptoms in autism spectrum disorders: a randomized controlled trial. Front. Psychiatry. 11, 550593. 10.3389/fpsyt.2020.550593 33101079 PMC7546872

[B137] Schwarz J. M. Bilbo S. D. (2012). Sex, glia, and development: interactions in health and disease. Hormones Behav. 62, 243–253. 10.1016/j.yhbeh.2012.02.018 22387107 PMC3374064

[B138] Sena L. A. Chandel N. S. (2012). Physiological roles of mitochondrial reactive oxygen species. Mol. Cell 48, 158–167. 10.1016/j.molcel.2012.09.025 23102266 PMC3484374

[B139] Seyedinia S. A. Tarahomi P. Abbarin D. Sedaghat K. Rashidy-Pour A. Yaribeygi H. (2023). Saffron and crocin ameliorate prenatal valproic acid-induced autistic-like behaviors and brain oxidative stress in the Male offspring rats. Metab. Brain Dis. 38, 2231–2241. 10.1007/s11011-023-01275-7 37566156

[B140] Shaaban S. Y. El Gendy Y. G. Mehanna N. S. El-Senousy W. M. El-Feki H. S. A. Saad K. (2018). The role of probiotics in children with autism spectrum disorder: a prospective, open-label study. Nutr. Neurosci. 21, 676–681. 10.1080/1028415X.2017.1347746 28686541

[B141] Sharma A. Parikh M. Shah H. Gandhi T. (2020). Modulation of Nrf2 by quercetin in doxorubicin-treated rats. Heliyon 6, e03803. 10.1016/j.heliyon.2020.e03803 32337383 PMC7177035

[B142] Shen Z. Wu W. Hazen S. L. (2000). Activated leukocytes oxidatively damage DNA, RNA, and the nucleotide pool through halide-dependent formation of hydroxyl radical. Biochemistry 39, 5474–5482. 10.1021/bi992809y 10820020

[B143] Siddiqui M. F. Elwell C. Johnson M. H. (2016). Mitochondrial dysfunction in autism spectrum disorders. Autism Open Access 6, 1–12. 10.4172/2165-7890.1000190 27928515 PMC5137782

[B144] Sies H. Berndt C. Jones D. P. (2017). Oxidative stress. Annu. Rev. Biochem. 86, 715–748. 10.1146/annurev-biochem-061516-045037 28441057

[B145] Takano T. (2015). Role of microglia in autism: recent advances. Dev. Neurosci. 37, 195–202. 10.1159/000398791 25998072

[B146] Tang G. Gutierrez Rios P. Kuo S.-H. Akman H. O. Rosoklija G. Tanji K. (2013). Mitochondrial abnormalities in temporal lobe of autistic brain. Neurobiol. Dis. 54, 349–361. 10.1016/j.nbd.2013.01.006 23333625 PMC3959772

[B147] Tebartz Van Elst L. Maier S. Fangmeier T. Endres D. Mueller G. T. Nickel K. (2014). Disturbed cingulate glutamate metabolism in adults with high-functioning autism spectrum disorder: evidence in support of the excitatory/inhibitory imbalance hypothesis. Mol. Psychiatry 19, 1314–1325. 10.1038/mp.2014.62 25048006

[B148] Tellone E. Galtieri A. Russo A. Giardina B. Ficarra S. (2015). Resveratrol: a focus on several neurodegenerative diseases. Oxidative Med. Cell. Longev. 2015, 1–14. 10.1155/2015/392169 26180587 PMC4477222

[B149] Theoharides T. C. Asadi S. Patel A. B. (2013). Focal brain inflammation and autism. J. Neuroinflammation 10, 815. 10.1186/1742-2094-10-46 23570274 PMC3626551

[B150] Theoharides T. C. Tsilioni I. Patel A. B. Doyle R. (2016). Atopic diseases and inflammation of the brain in the pathogenesis of autism spectrum disorders. Transl. Psychiatry 6, e844. 10.1038/tp.2016.77 27351598 PMC4931610

[B151] Urakubo A. Jarskog L. F. Lieberman J. A. Gilmore J. H. (2001). Prenatal exposure to maternal infection alters cytokine expression in the placenta, amniotic fluid, and fetal brain. Schizophrenia Res. 47, 27–36. 10.1016/S0920-9964(00)00032-3 11163542

[B152] Valiente-Pallejà A. Torrell H. Muntané G. Cortés M. J. Martínez-Leal R. Abasolo N. (2018). Genetic and clinical evidence of mitochondrial dysfunction in autism spectrum disorder and intellectual disability. Hum. Mol. Genet. 27, 891–900. 10.1093/hmg/ddy009 29340697

[B153] Vargas D. L. Nascimbene C. Krishnan C. Zimmerman A. W. Pardo C. A. (2005). Neuroglial activation and neuroinflammation in the brain of patients with autism. Ann. Neurology 57, 67–81. 10.1002/ana.20315 15546155

[B154] Vasconcelos C. Perry I. S. Gottfried C. Riesgo R. Castro K. (2025). Folic acid and autism: updated evidences. Nutr. Neurosci. 28, 273–307. 10.1080/1028415X.2024.2367855 38968136

[B155] Veselinović A. Petrović S. Žikić V. Subotić M. Jakovljević V. Jeremić N. (2021). Neuroinflammation in autism and supplementation based on Omega-3 polyunsaturated fatty acids: a narrative review. Medicina 57, 893. 10.3390/medicina57090893 34577816 PMC8464922

[B156] Wang X. Yang J. Zhang H. Yu J. Yao Z. (2019). Oral probiotic administration during pregnancy prevents autism‐related behaviors in offspring induced by maternal immune activation *via* anti‐inflammation in mice. Autism Res. 12, 576–588. 10.1002/aur.2079 30681777

[B157] Wang J. Zhu H. Wang K. Yang Z. Liu Z. (2020). Protective effect of quercetin on rat testes against cadmium toxicity by alleviating oxidative stress and autophagy. Environ. Sci. Pollut. Res. 27, 25278–25286. 10.1007/s11356-020-08947-2 32347499

[B158] Wang Y. Li N. Yang J.-J. Zhao D.-M. Chen B. Zhang G.-Q. (2020). Probiotics and fructo-oligosaccharide intervention modulate the microbiota-gut brain axis to improve autism spectrum reducing also the hyper-serotonergic state and the dopamine metabolism disorder. Pharmacol. Res. 157, 104784. 10.1016/j.phrs.2020.104784 32305492

[B159] Williams P. G. Sears L. Watson W. H. Gunaratnam B. Feygin Y. Wright S. P. (2025). Glutathione, vitamin C, and cysteine use in autistic children with disruptive behavior: a double-blind, placebo-controlled crossover pilot study. J. Dev. Behav. Pediatr. 46, e17–e24. 10.1097/DBP.0000000000001334 39960783

[B160] Yang C.-J. Liu C.-L. Sang B. Zhu X.-M. Du Y.-J. (2015). The combined role of serotonin and interleukin-6 as biomarker for autism. Neuroscience 284, 290–296. 10.1016/j.neuroscience.2014.10.011 25453766

[B161] Yang J. Fu X. Liao X. Li Y. (2020). Nrf2 activators as dietary phytochemicals against oxidative stress, inflammation, and mitochondrial dysfunction in autism spectrum disorders: a systematic review. Front. Psychiatry 11, 561998. 10.3389/fpsyt.2020.561998 33329102 PMC7714765

[B162] Zarezadeh M. Mahmoudinezhad M. Hosseini B. Khorraminezhad L. Razaghi M. Alvandi E. (2023). Dietary pattern in autism increases the need for probiotic supplementation: a comprehensive narrative and systematic review on oxidative stress hypothesis. Clin. Nutr. 42, 1330–1358. 10.1016/j.clnu.2023.06.014 37418842

[B163] Zeng X. Fan L. Li M. Qin Q. Pang X. Shi S. (2024). Resveratrol regulates Thoc5 to improve maternal immune activation-induced autism-like behaviors in adult mouse offspring. J. Nutr. Biochem. 129, 109638. 10.1016/j.jnutbio.2024.109638 38583499

[B164] Zhong H. Xiao R. Ruan R. Liu H. Li X. Cai Y. (2020). Neonatal curcumin treatment restores hippocampal neurogenesis and improves autism-related behaviors in a mouse model of autism. Psychopharmacology 237, 3539–3552. 10.1007/s00213-020-05634-5 32803366

[B165] Zimmerman A. W. Connors S. L. Matteson K. J. Lee L.-C. Singer H. S. Castaneda J. A. (2007). Maternal antibrain antibodies in autism. Brain, Behav. Immun. 21, 351–357. 10.1016/j.bbi.2006.08.005 17029701

